# The artificial intelligence-based model ANORAK improves histopathological grading of lung adenocarcinoma

**DOI:** 10.1038/s43018-023-00694-w

**Published:** 2024-01-10

**Authors:** Xiaoxi Pan, Khalid AbdulJabbar, Jose Coelho-Lima, Anca-Ioana Grapa, Hanyun Zhang, Alvin Ho Kwan Cheung, Juvenal Baena, Takahiro Karasaki, Claire Rachel Wilson, Marco Sereno, Selvaraju Veeriah, Sarah J. Aitken, Allan Hackshaw, Andrew G. Nicholson, Mariam Jamal-Hanjani, TRACERx Consortium, John Le Quesne, Sam M. Janes, Anne-Marie Hacker, Abigail Sharp, Sean Smith, Harjot Kaur Dhanda, Kitty Chan, Camilla Pilotti, Rachel Leslie, David Chuter, Mairead MacKenzie, Serena Chee, Aiman Alzetani, Eric Lim, Paulo De Sousa, Simon Jordan, Alexandra Rice, Hilgardt Raubenheimer, Harshil Bhayani, Lyn Ambrose, Anand Devaraj, Hema Chavan, Sofina Begum, Silviu I. Buderi, Daniel Kaniu, Mpho Malima, Sarah Booth, Nadia Fernandes, Pratibha Shah, Chiara Proli, Madeleine Hewish, Sarah Danson, Michael J. Shackcloth, Lily Robinson, Peter Russell, Kevin G. Blyth, Andrew Kidd, Alan Kirk, Mo Asif, Rocco Bilancia, Nikos Kostoulas, Mathew Thomas, Craig Dick, Jason F. Lester, Amrita Bajaj, Apostolos Nakas, Azmina Sodha-Ramdeen, Mohamad Tufail, Molly Scotland, Rebecca Boyles, Sridhar Rathinam, Dean A. Fennell, Claire Wilson, Domenic Marrone, Sean Dulloo, Gurdeep Matharu, Jacqui A. Shaw, Joan Riley, Lindsay Primrose, Ekaterini Boleti, Heather Cheyne, Mohammed Khalil, Shirley Richardson, Tracey Cruickshank, Gillian Price, Keith M. Kerr, Sarah Benafif, Dionysis Papadatos-Pastos, James Wilson, Tanya Ahmad, Jack French, Kayleigh Gilbert, Babu Naidu, Akshay J. Patel, Aya Osman, Christer Lacson, Gerald Langman, Helen Shackleford, Madava Djearaman, Salma Kadiri, Gary Middleton, Angela Leek, Jack Davies Hodgkinson, Nicola Totten, Angeles Montero, Elaine Smith, Eustace Fontaine, Felice Granato, Juliette Novasio, Kendadai Rammohan, Leena Joseph, Paul Bishop, Rajesh Shah, Stuart Moss, Vijay Joshi, Philip Crosbie, Antonio Paiva-Correia, Anshuman Chaturvedi, Lynsey Priest, Pedro Oliveira, Fabio Gomes, Kate Brown, Mathew Carter, Colin R. Lindsay, Fiona H. Blackhall, Matthew G. Krebs, Yvonne Summers, Alexandra Clipson, Jonathan Tugwood, Alastair Kerr, Dominic G. Rothwell, Caroline Dive, Hugo J. W. L. Aerts, Roland F. Schwarz, Tom L. Kaufmann, Peter Van Loo, Gareth A. Wilson, Rachel Rosenthal, Andrew Rowan, Chris Bailey, Claudia Lee, Emma Colliver, Katey S. S. Enfield, Mark S. Hill, Mihaela Angelova, Oriol Pich, Michelle Leung, Alexander M. Frankell, Crispin T. Hiley, Emilia L. Lim, Haoran Zhai, Maise Al Bakir, Nicolai J. Birkbak, Olivia Lucas, Ariana Huebner, Clare Puttick, Kristiana Grigoriadis, Michelle Dietzen, Dhruva Biswas, Foteini Athanasopoulou, Sophia Ward, Jonas Demeulemeester, Carla Castignani, Elizabeth Larose Cadieux, Judit Kisistok, Mateo Sokac, Zoltan Szallasi, Miklos Diossy, Roberto Salgado, Aengus Stewart, Alastair Magness, Clare E. Weeden, Dina Levi, Eva Grönroos, Imran Noorani, Jacki Goldman, Mickael Escudero, Philip Hobson, Roberto Vendramin, Stefan Boeing, Tamara Denner, Vittorio Barbè, Wei-Ting Lu, William Hill, Yutaka Naito, Zoe Ramsden, George Kassiotis, Angela Dwornik, Angeliki Karamani, Benny Chain, David R. Pearce, Despoina Karagianni, Felip Gálvez-Cancino, Georgia Stavrou, Gerasimos Mastrokalos, Helen L. Lowe, Ignacio Garcia Matos, James L. Reading, John A. Hartley, Kayalvizhi Selvaraju, Kezhong Chen, Leah Ensell, Mansi Shah, Maria Litovchenko, Olga Chervova, Piotr Pawlik, Robert E. Hynds, Samuel Gamble, Seng Kuong Anakin Ung, Supreet Kaur Bola, Victoria Spanswick, Yin Wu, Othman Al-Sawaf, Thomas Patrick Jones, Stephan Beck, Miljana Tanic, Teresa Marafioti, Elaine Borg, Mary Falzon, Reena Khiroya, Antonia Toncheva, Christopher Abbosh, Corentin Richard, Cristina Naceur-Lombardelli, Francisco Gimeno-Valiente, Krupa Thakkar, Mariana Werner Sunderland, Monica Sivakumar, Nnennaya Kanu, Paulina Prymas, Sadegh Saghafinia, Sharon Vanloo, Jie Min Lam, Wing Kin Liu, Abigail Bunkum, Sonya Hessey, Simone Zaccaria, Carlos Martínez-Ruiz, James R. M. Black, Kerstin Thol, Robert Bentham, Kevin Litchfield, Nicholas McGranahan, Sergio A. Quezada, Martin D. Forster, Siow Ming Lee, Javier Herrero, Emma Nye, Richard Kevin Stone, Jerome Nicod, Jayant K. Rane, Karl S. Peggs, Kevin W. Ng, Krijn Dijkstra, Matthew R. Huska, Emilie Martinoni Hoogenboom, Fleur Monk, James W. Holding, Junaid Choudhary, Kunal Bhakhri, Marco Scarci, Pat Gorman, Robert C. M. Stephens, Yien Ning Sophia Wong, Zoltan Kaplar, Steve Bandula, Thomas B. K. Watkins, Catarina Veiga, Gary Royle, Charles-Antoine Collins-Fekete, Francesco Fraioli, Paul Ashford, Alexander James Procter, Asia Ahmed, Magali N. Taylor, Arjun Nair, David Lawrence, Davide Patrini, Neal Navani, Ricky M. Thakrar, Charles Swanton, Yinyin Yuan, John Le Quesne, David A. Moore

**Affiliations:** 1 Centre for Evolution and Cancer, The Institute of Cancer Research, London, UK; 2 Division of Molecular Pathology, The Institute of Cancer Research, London, UK; 3 Medical Research Council Toxicology Unit, University of Cambridge, Cambridge, UK; 4 Department of Histopathology, Cambridge University Hospitals NHS Foundation Trust, Cambridge, UK; 5 Cancer Evolution and Genome Instability Laboratory, The Francis Crick Institute, London, UK; 6 Leicester Cancer Research Centre, University of Leicester, Leicester, UK; 7 Cancer Research UK Lung Cancer Centre of Excellence, University College London Cancer Institute, London, UK; 8 Hope Against Cancer and Leicester Experimental Cancer Medicine Centre, Leicester, UK; 9 Institute for Lung Health, NIHR Leicester Biomedical Research Centre, Leicester, UK; 10 Cancer Research UK & UCL Cancer Trials Centre, London, UK; 11 Department of Histopathology, Royal Brompton and Harefield Hospitals, Guy’s and St Thomas’ NHS Foundation Trust, London, UK; 12 National Heart and Lung Institute, Imperial College London, London, UK; 13 Cancer Metastasis Laboratory, University College London Cancer Institute, London, UK; 14 Department of Medical Oncology, University College London Hospitals NHS Foundation Trust, London, UK; 15 Molecular Pathology, School of Cancer Sciences, University of Glasgow, Glasgow, UK; 16 Cancer Research UK Beatson Institute of Cancer Research, Glasgow, UK; 17 NHS Greater Glasgow and Clyde, Glasgow, UK; 18 Department of Cellular Pathology, University College London Hospitals, London, UK; 19 Present Address: Department of Translational Molecular Pathology, The University of Texas MD Anderson Cancer Center, Houston, TX USA; 20 Present Address: AstraZeneca Computational Pathology, Munich, Germany; 21 Lungs for Living Research Centre, UCL Respiratory, Department of Medicine, University College London, London, UK; 22 Independent Cancer Patient’s Voice, London, UK; 23 University Hospital Southampton NHS Foundation Trust, Southampton, UK; 24 Academic Division of Thoracic Surgery, Imperial College London, London, UK; 25 Royal Brompton and Harefield Hospitals, Guy’s and St Thomas’ NHS Foundation Trust, London, UK; 26 Royal Surrey Hospital, Royal Surrey Hospitals NHS Foundation Trust, Guildford, UK; 27 University of Surrey, Guildford, UK; 28 Sheffield Teaching Hospitals NHS Foundation Trust, Sheffield, UK; 29 Liverpool Heart and Chest Hospital, Liverpool, UK; 30 Princess Alexandra Hospital, The Princess Alexandra Hospital NHS Trust, Harlow, UK; 31 School of Cancer Sciences, University of Glasgow, Glasgow, UK; 32 Beatson Institute for Cancer Research, University of Glasgow, Glasgow, UK; 33 Queen Elizabeth University Hospital, Glasgow, UK; 34 Institute of Infection, Immunity & Inflammation, University of Glasgow, Glasgow, UK; 35 Golden Jubilee National Hospital, Clydebank, UK; 36 Singleton Hospital, Swansea Bay University Health Board, Swansea, UK; 37 University Hospitals of Leicester NHS Trust, Leicester, UK; 38 University of Leicester, Leicester, UK; 39 Cancer Research Centre, University of Leicester, Leicester, UK; 40 Royal Free London NHS Foundation Trust, London, UK; 41 Aberdeen Royal Infirmary NHS Grampian, Aberdeen, UK; 42 Department of Medical Oncology, Aberdeen Royal Infirmary NHS Grampian, Aberdeen, UK; 43 University of Aberdeen, Aberdeen, UK; 44 Department of Pathology, Aberdeen Royal Infirmary NHS Grampian, Aberdeen, UK; 45 Department of Oncology, University College London Hospitals, London, UK; 46 The Whittington Hospital NHS Trust, London, UK; 47 Birmingham Acute Care Research Group, Institute of Inflammation and Ageing, University of Birmingham, Birmingham, UK; 48 University Hospital Birmingham NHS Foundation Trust, Birmingham, UK; 49 Artificial Intelligence in Medicine AIM Program, Mass General Brigham, Harvard Medical School, Boston, MA USA; 50 Institute of Immunology and Immunotherapy, University of Birmingham, Birmingham, UK; 51 Manchester Cancer Research Centre Biobank, Manchester, UK; 52 Wythenshawe Hospital, Manchester University NHS Foundation Trust, Manchester, UK; 53 Division of Infection, Immunity and Respiratory Medicine, University of Manchester, Manchester, UK; 54 Cancer Research UK Lung Cancer Centre of Excellence, University of Manchester, Manchester, UK; 55 Manchester University NHS Foundation Trust, Manchester, UK; 56 The Christie NHS Foundation Trust, Manchester, UK; 57 Division of Cancer Sciences, The University of Manchester and The Christie NHS Foundation Trust, Manchester, UK; 58 Cancer Research UK Manchester Institute Cancer Biomarker Centre, University of Manchester, Manchester, UK; 59 Department of Radiation Oncology, Brigham and Women’s Hospital, Dana-Farber Cancer Institute, Harvard Medical School, Boston, MA USA; 60 Radiology and Nuclear Medicine, CARIM & GROW, Maastricht University, Maastricht, the Netherlands; 61 Institute for Computational Cancer Biology, Center for Integrated Oncology, Cancer Research Center Cologne Essen, Faculty of Medicine and University Hospital Cologne, University of Cologne, Cologne, Germany; 62 Berlin Institute for the Foundations of Learning and Data, Berlin, Germany; 63 Berlin Institute for Medical Systems Biology, Max Delbrück Center for Molecular Medicine in the Helmholtz Association, Berlin, Germany; 64 Department of Genetics, The University of Texas MD Anderson Cancer Center, Houston, TX USA; 65 Department of Genomic Medicine, The University of Texas MD Anderson Cancer Center, Houston, TX USA; 66 Cancer Genomics Laboratory, The Francis Crick Institute, London, UK; 67 Cancer Genome Evolution Research Group, Cancer Research UK Lung Cancer Centre of Excellence, University College London Cancer Institute, London, UK; 68 Department of Molecular Medicine, Aarhus University Hospital, Aarhus, Denmark; 69 Department of Clinical Medicine, Aarhus University, Aarhus, Denmark; 70 Bioinformatics Research Centre, Aarhus University, Aarhus, Denmark; 71 Computational Cancer Genomics Research Group, University College London Cancer Institute, London, UK; 72 University College London Hospitals, London, UK; 73 Bill Lyons Informatics Centre, University College London Cancer Institute, London, UK; 74 Advanced Sequencing Facility, The Francis Crick Institute, London, UK; 75 Integrative Cancer Genomics Laboratory, Department of Oncology, KU Leuven, Leuven, Belgium; 76 VIB-KU Leuven Center for Cancer Biology, Leuven, Belgium; 77 Medical Genomics, University College London Cancer Institute, London, UK; 78 Danish Cancer Society Research Center, Copenhagen, Denmark; 79 Computational Health Informatics Program, Boston Children’s Hospital, Boston, MA USA; 80 Department of Bioinformatics, Semmelweis University, Budapest, Hungary; 81 Department of Physics of Complex Systems, ELTE Eötvös Loránd University, Budapest, Hungary; 82 Department of Pathology, Ziekenhuis aan de Stroom Hospitals, Antwerp, Belgium; 83 Division of Research, Peter MacCallum Cancer Centre, Melbourne, Victoria Australia; 84 The Francis Crick Institute, London, UK; 85 Department of Infectious Disease, Faculty of Medicine, Imperial College London, London, UK; 86 University College London Cancer Institute, London, UK; 87 Department I of Internal Medicine, University Hospital of Cologne, Cologne, Germany; 88 Experimental Oncology, Institute for Oncology and Radiology of Serbia, Belgrade, Serbia; 89 Tumour Immunogenomics and Immunosurveillance Laboratory, University College London Cancer Institute, London, UK; 90 Immune Regulation and Tumour Immunotherapy Group, Cancer Immunology Unit, Research Department of Haematology, University College London Cancer Institute, London, UK; 91 Experimental Histopathology, The Francis Crick Institute, London, UK; 92 Department of Haematology, University College London Hospitals, London, UK; 93 Cancer Immunology Unit, Research Department of Haematology, University College London Cancer Institute, London, UK; 94 Retroviral Immunology Group, The Francis Crick Institute, London, UK; 95 Department of Molecular Oncology and Immunology, The Netherlands Cancer Institute, Amsterdam, the Netherlands; 96 Oncode Institute, Utrecht, the Netherlands; 97 Bioinformatics and Systems Biology, Method Development and Research Infrastructure, Robert Koch Institute, Berlin, Germany; 98 Department of Medical Physics and Biomedical Engineering, Centre for Medical Image Computing, London, UK; 99 Department of Medical Physics and Bioengineering, University College London Cancer Institute, London, UK; 100 Department of Medical Physics and Biomedical Engineering, University College London, London, UK; 101 Institute of Nuclear Medicine, Division of Medicine, University College London, London, UK; 102 Institute of Structural and Molecular Biology, University College London, London, UK; 103 Department of Radiology, University College London Hospitals, London, UK; 104 University College London Respiratory, Department of Medicine, University College London, London, UK; 105 Department of Thoracic Surgery, University College London Hospital NHS Trust, London, UK; 106 Lungs for Living Research Centre, University College London Respiratory, University College London, London, UK; 107 Department of Thoracic Medicine, University College London Hospitals, London, UK

**Keywords:** Non-small-cell lung cancer, Machine learning, Image processing

## Abstract

The introduction of the International Association for the Study of Lung Cancer grading system has furthered interest in histopathological grading for risk stratification in lung adenocarcinoma. Complex morphology and high intratumoral heterogeneity present challenges to pathologists, prompting the development of artificial intelligence (AI) methods. Here we developed ANORAK (pyrAmid pooliNg crOss stReam Attention networK), encoding multiresolution inputs with an attention mechanism, to delineate growth patterns from hematoxylin and eosin-stained slides. In 1,372 lung adenocarcinomas across four independent cohorts, AI-based grading was prognostic of disease-free survival, and further assisted pathologists by consistently improving prognostication in stage I tumors. Tumors with discrepant patterns between AI and pathologists had notably higher intratumoral heterogeneity. Furthermore, ANORAK facilitates the morphological and spatial assessment of the acinar pattern, capturing acinus variations with pattern transition. Collectively, our AI method enabled the precision quantification and morphology investigation of growth patterns, reflecting intratumoral histological transitions in lung adenocarcinoma.

## Abstract (web summary)

Yuan and colleagues developed an artificial intelligence-based method to derive growth patterns and morphological features from hematoxylin and eosin-stained slides of lung adenocarcinoma samples, for improved tumor grading and patient prognostication.

## Main

**Extended Data Fig. 1:**
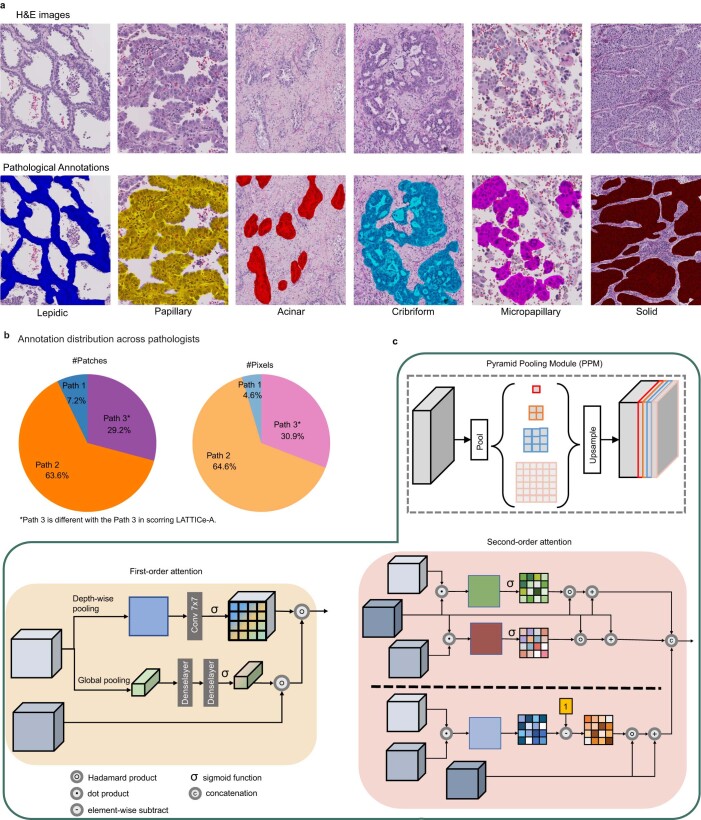
Precise pathological annotations for training and sub-modules of the developed deep learning model (ANORAK). **a**. Examples illustrating morphologically distinct growth patterns in lung adenocarcinoma. **b**. Distribution of annotations regarding the number of patches and pixels. **c**. Detailed architectures of sub-modules developed for the AI method.

Lung adenocarcinoma (LUAD), the most common type of non-small cell lung cancer, is histologically characterized by distinct growth patterns: lepidic, papillary, acinar, cribriform, micropapillary and solid^1^ (Extended Data Fig. 1a). The proposed International Association for the Study of Lung Cancer (IASLC) grading system, based on a combination of the predominant growth pattern and high-grade patterns (cribriform, micropapillary and solid) within individual tumors, is highly prognostic^2^. However, there is interobserver variability among pathologists due to the challenges of consistently defining, recognizing and quantifying the wide spectrum of growth patterns^3^. This variability particularly affects differentiating lepidic, papillary and acinar patterns^2,4^, as well as the estimated proportion of high-grade patterns in non-high-grade pattern-predominant tumors^2,5^. Accurate quantification is challenging when there are multiple admixed growth patterns across several histological sections, as is the case in most LUADs. This challenge is compounded by the difficulty of defining the cutoff between different patterns where they represent a spectrum of histological appearances^6^. This poses challenges for accurate prognostic inference and reproducibility in clinical studies.

Computer-assisted approaches powered by artificial intelligence (AI) have been widely applied to histological image analysis^7–11^. While some studies have applied deep learning models to LUAD growth pattern classification^12,13^, automated IASLC grading by AI methods is yet to be explored. Moreover, previous deep learning methods were mainly based on patch-wise classification that predicts a histological subtype for each patch, overlooking the detailed morphological structure of patterns. To capture the distinct pattern morphology, we developed an AI method based on pixel-wise classification to segment growth pattern islands and automate the IASLC grading for risk stratification and outcome prediction.

**Fig. 1:**
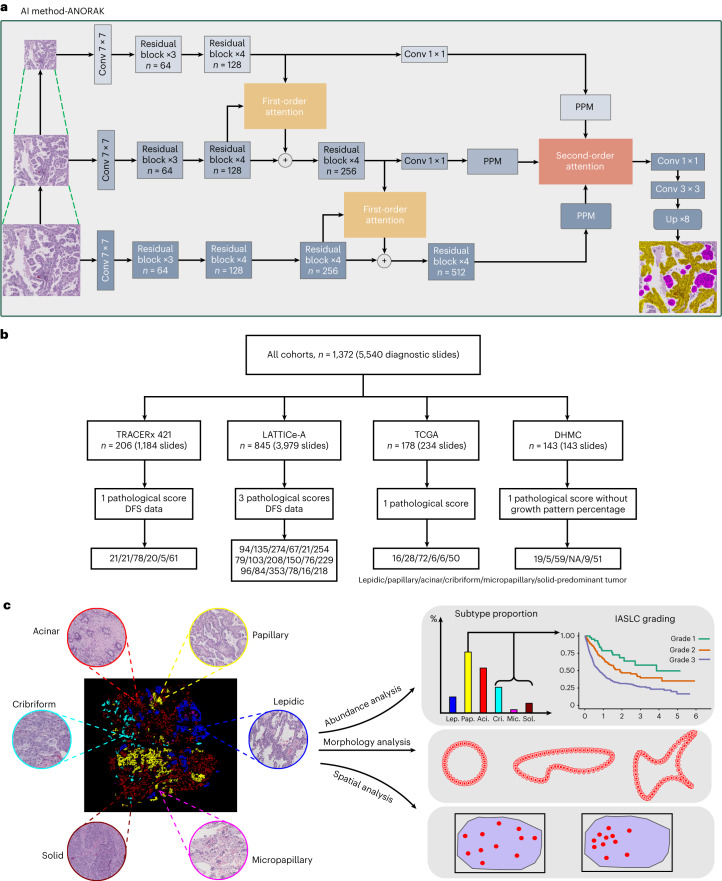
Proposed computational pipeline for precision mapping and spatial heterogeneity analyses. **a**, The deep learning network architecture for growth pattern segmentation integrating inputs over multiple spatial resolutions and delivering pixel-wise delineations. **b**, Overview of all the cohorts and available data. **c**, Downstream analyses enabled by the AI method, including abundance quantification, risk stratification and morphological and spatial heterogeneity analyses. PPM, Pyramid Pooling Module; Lep., lepidic; Pap., papillary; Aci., acinar; Cri., cribriform; Mic., micropapillary; Sol., solid; NA, not applicable. Source data

In this study, we developed an AI method to segment LUAD growth patterns at the pixel level using hematoxylin and eosin (H&E) whole-slide images (WSIs) (Fig. 1a and Extended Data Fig. 1b,c) and applied it to 5,540 diagnostic slides from 1,372 cases, spanning four cohorts: TRAcking non-small cell lung Cancer Evolution through therapy (Rx) (TRACERx); Leicester Archival Thoracic Tumor Investigatory Cohort-Adenocarcinoma (LATTICe-A); The Cancer Genome Atlas (TCGA) LUAD; and Dartmouth Lung Cancer Histology Dataset (DHMC) (Fig. 1b). The growth pattern proportions, predominant pattern and IASLC grading of a tumor can be derived automatically based on growth pattern mapping (Fig. 1c). This pixel-wise segmentation method also revealed the morphological properties of growth patterns and enabled analysis of the degree of spatial heterogeneity, highlighting its advantages over patch-wise classification algorithms.

## Results

### A hierarchical AI model for growth pattern quantification

To spatially map complex growth patterns in LUAD, we developed ANORAK (pyrAmid pooliNg crOss stReam Attention networK), which encodes cross-stream interactions using a multi-order attention mechanism within convolutional neural networks^14^ (Fig. 1a and Extended Data Fig. 1b,c). Moreover, a pyramid pooling module (PPM)^15^ distributed global contextual information of growth patterns to guide high-level feature learning. ANORAK was trained on data annotated from 49 WSIs in the TRACERx 100 cohort (Extended Data Fig. 1a) by three thoracic subspeciality pathologists (Extended Data Fig. 1b), and validated on a total of 5,540 WSIs from 1,372 LUAD tumors across four cohorts (Fig. 1b and Table 1). This model enabled precision mapping of diverse growth patterns at pixel-level resolution, thereby facilitating automated grading and analysis of morphological intratumoral heterogeneity (Fig. 1c).

**Table 1:** Patient demographics (all cohorts)

Characteristic	TRACERx 421	LATTICe-A	TCGA LUAD	DHMC
Number of patients (diagnostic slides)	206 (1,184)	845 (3,979)	178 (234)	143 (143)
Pathological score available	1	3	1	1
Age, mean (minimum, maximum)	68.43 (37, 92)	67.66 (31, 86)	65.48 (42, 85)	–
Sex, *n* (%)				
Female	111 (53.88)	444 (52.54)	102 (57.30)	–
Male	95 (46.12)	401 (47.46)	76 (42.70)	–
Tumor stage, *n* (%)				
I	108 (52.43)	337 (39.88)	92 (51.69)	–
II	54 (26.21)	202 (23.91)	40 (22.47)	–
III	44 (21.36)	190 (22.49)	33 (18.54)	–
IV	0 (0)	0 (0)	12 (6.74)	–
Not applicable	0 (0)	116 (13.73)	1 (0.56)	–
Smoking status, *n* (%)				
Current smoker	88 (42.72)	259 (30.65)	–	–
Ex-smoker	101 (49.03)	419 (49.59)	–	–
Never smoker	17 (8.25)	64 (7.57)	–	–
Not applicable	–	103 (12.19)		
Adjuvant treatment, *n* (%)				
Yes	64 (31.07)	134 (15.86)	–	–
No	142 (68.93)	711 (84.14)	–	–
Type of surgery, *n* (%)				
Lobectomy or greater	180 (83.98)	640 (70.53)	–	–
Sublobar resection	26 (16.02)	89 (29.47)	–	–

**Fig. 2:**
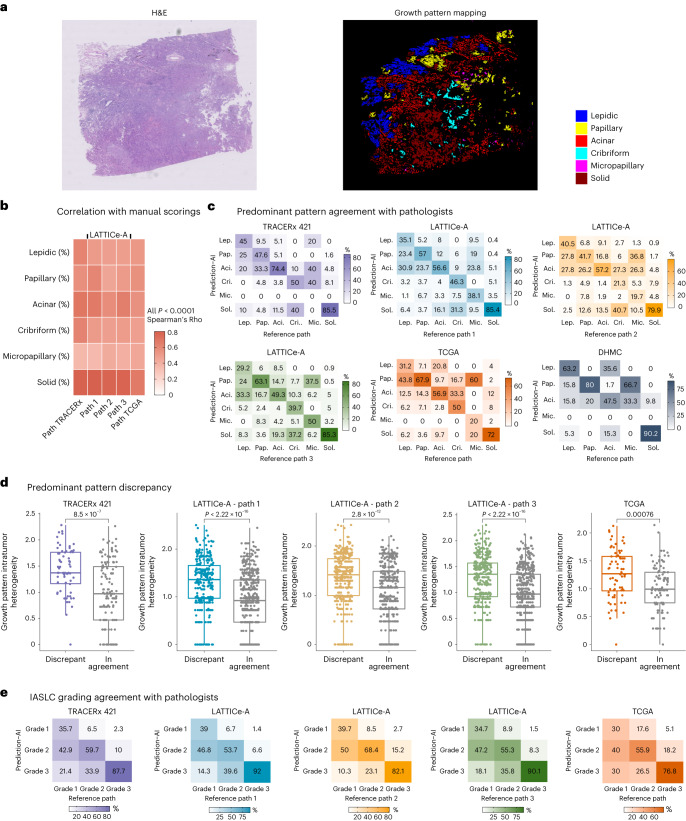
Performance of AI in the prediction and quantification of growth patterns. **a**, Segmentation example generated by ANORAK. **b**, Correlations of growth pattern proportions at the tumor level between AI and pathologists. Growth pattern proportions were not available in the DHMC cohort; thus, plots relevant to proportions were not illustrated for the DHMC (same in **d** and **e**). *P* values were corrected for multiple comparisons using the Benjamini–Hochberg method. **c**, Performance comparison with pathologists in predicting the predominant pattern per case (the cribriform predominant slide per tumor was not available in the DHMC cohort). **d**, Growth pattern intratumoral heterogeneity substantially contributed to the discrepancy between AI and pathologists (TRACERx 421, *P* = 8.467 × 10^−7^, *n* = 206; LATTICe-A, *P*1 < 2.22 × 10^−16^, *P*2 = 2.816 × 10^−12^, *P*3 < 2.22 × 10^−16^, *n* = 845; TCGA, *P* = 0.0007632, *n* = 177). Each *P* value was calculated using a two-sided Wilcoxon rank-sum test and not adjusted for multiple comparisons. The median value is indicated by a thick horizontal line; the first and third quartiles are represented by the box edges; the whiskers indicate 1.5× the interquartile range. **e**, Performance comparison with pathologists in the prediction of IASLC grading per case. Source data

**Extended Data Fig. 2:**
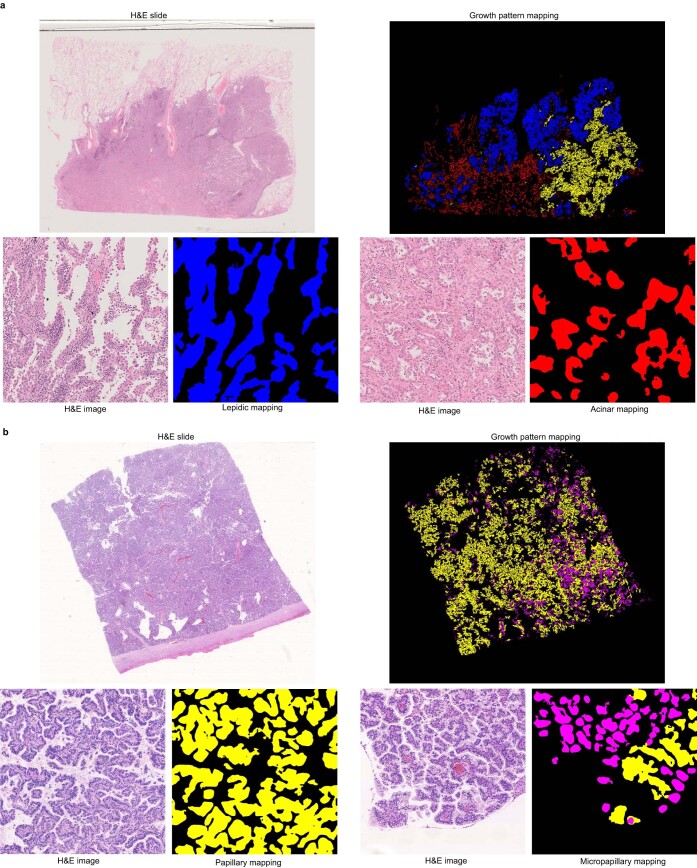
Segmentation performance. **a**,**b**. Segmentations generated by AI at low-power and high-power resolutions, deposited in 10.6084/m9.figshare.24599796.

**Extended Data Fig. 3:**
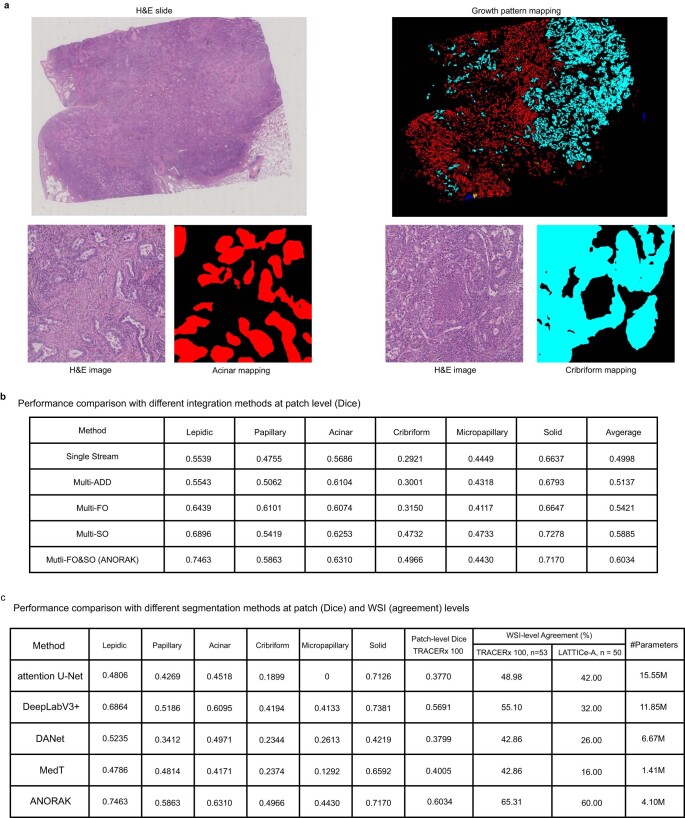
Segmentation performance and intra- and inter-comparisons. **a**. Segmentations generated by AI at low-power and high-power resolutions, deposited in 10.6084/m9.figshare.24599796. **b**. Comparison of segmentation and prediction performance for ablation experiments. **c**. Comparison of segmentation and prediction performance with other methods.

ANORAK generated promising outputs for growth pattern segmentation (Fig. 2a and Extended Data Figs. 2a,b and 3a). To validate the effectiveness of the developed model, we conducted the ablation study at the patch level (Extended Data Fig. 3b). Overall, multi-stream variants were more promising than single-stream ones, gaining an advantage by gathering different types of features. Moreover, methods with attention modules (multi-FO, multi-SO, ANORAK) achieved better overall performance, implying that the attention techniques came into effect. Specifically, first-order attention (multi-FO) improved performance by around 3% compared to the adding fashion (multi-ADD), while second-order attention (multi-SO) showed an approximate 5% improvement when compared to multi-FO. This suggested that high-level feature interactions across streams could be more effective than merging at low-level feature learning, highlighting the importance of high-level features in semantic segmentation^15,16^. The proposed model adopted both first-order and second-order attention modules, enhancing the overall performance with notable improvements. To compare this with existing methods, ANORAK outperformed several widely used approaches in semantic segmentation, including attention U-Net^17^, DeepLabv3+ (ref. ^18^), DANet^19^ and MedT^20^, for growth pattern subtypes (0.4430–0.7463; Extended Data Fig. 3c) except for solid pattern (0.7170), which was lower than DeepLabv3+ (0.7381). ANORAK also achieved overall promising performance at the patch-level and WSI-level evaluations (patch-Dice: ANORAK: 0.6034, other methods: 0.3770–0.5691; WSI agreement: ANORAK: 60.00–65.31%, other methods: 16–48.98%; Extended Data Fig. 3c). Furthermore, the parameters of ANORAK are 4.10 million, that is, more lightweight than other convolutional models (6.67–15.55 million; Extended Data Fig. 3c). Taken together, the proposed model may have advantages in performance and computing over other methods.

**Extended Data Fig. 4:**
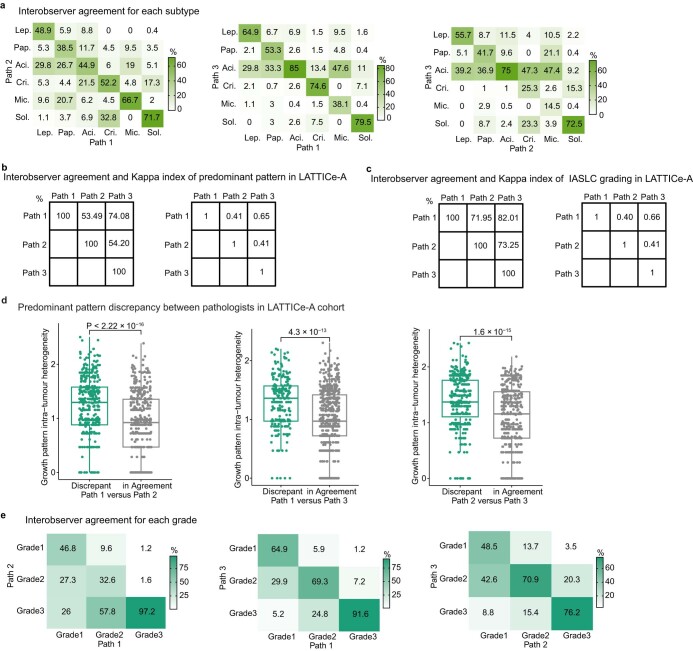
Inter-pathologists comparison for predominant pattern and IASLC grading in LATTICe-A. **a**. Interobserver agreement of each pattern. **b**, **c**. Interobserver agreement of predominant pattern and IASLC grading at tumor level. **d**. Growth pattern intra-tumoral heterogeneity substantially contributed to the discrepancy between pathologists (n = 845 each, P1 < 2.22 × 10^−16^, P2 = 4.323× 10^−13^, P3 = 1.589 × 10^−15^). P value was calculated using a two-sided Wilcoxon rank-sum test and not adjusted for the multiple comparisons. The median value is indicated by a thick horizontal line; the first and third quartiles are represented by box edges; whiskers indicate 1.5 times interquartile range. **e**. Interobserver agreement of each grade.

In all four cohorts, AI-predicted growth pattern proportions were highly correlated with the pathologists’ estimates (Fig. 2b and Supplementary Table 1), notably for the solid pattern (TRACERx 421, Spearman’s rho = 0.79; LATTICe-A correlations against each pathologist’s scoring, rho1 = 0.80, rho2 = 0.77, rho3 = 0.78; TCGA, rho = 0.67). The lowest correlations were observed for the micropapillary pattern (rho = 0.35–0.44 across three cohorts), which was also the pattern with the lowest interobserver agreement (LATTICe-A, 14.5–66.7%, average 39.8%; Extended Data Fig. 4a). When tumors were grouped according to their predominant pattern, the overall agreement rates between AI-predicted and manual scoring ranged between 50.18% and 67.96% (Supplementary Table 2 and Fig. 2c) across four cohorts. This is lower than the interobserver rates in LATTICe-A (53.49–74.08%; Extended Data Fig. 4b) but consistent with the known level of agreement between pathologists in previous studies (≥51.7%)^3,13^. The kappa statistics suggested a moderate agreement between AI and pathologists as well as inter-pathologists for predominant pattern assessment (averaged kappa index of AI-pathologist in four cohorts = 0.46; inter-AI-pathologists in LATTICe-A = 0.46; inter-pathologists in LATTICe-A = 0.49; Supplementary Table 3 and Extended Data Fig. 4b). Likewise, the overall agreement rates of AI-based grading according to the IASLC guidelines (AI grading hereafter) (65.73–76.80%; Supplementary Table 2 and Fig. 2e) were lower than the rates between pathologists in LATTICe-A (71.95–82.01%; Extended Data Fig. 4c,e), but the kappa statistics indicated a moderate agreement with manual grading, comparable with interobserver agreement (averaged kappa index of AI-pathologist in four cohorts = 0.47; inter-AI-pathologists in LATTICe-A = 0.50; inter-pathologists in LATTICe-A = 0.50; Supplementary Table 3 and Extended Data Fig. 4c). Interestingly, tumors with discrepant classification between AI and manual scoring had a notably higher intratumoral heterogeneity in growth pattern composition, measured using the Shannon diversity index based on pathological scores, compared to tumors concordant between AI and manual scoring (TRACERx 421, *P* = 8.5 × 10^−7^; LATTICe-A, *P*1 < 2.22 × 10^−16^, *P*2 = 2.8 × 10^−12^, *P*3 < 2.22 × 10^−16^; TCGA, *P* = 0.00076; Fig. 2d). A consistent trend was observed between discrepant and agreement classifications assessed by pathologists in LATTICe-A (*P* < 2.22 × 10^−16^, 4.3 × 10^−13^, 1.6 × 10^−15^; Extended Data Fig. 4d).

### AI grading consistently improves patient risk stratification

**Fig. 3:**
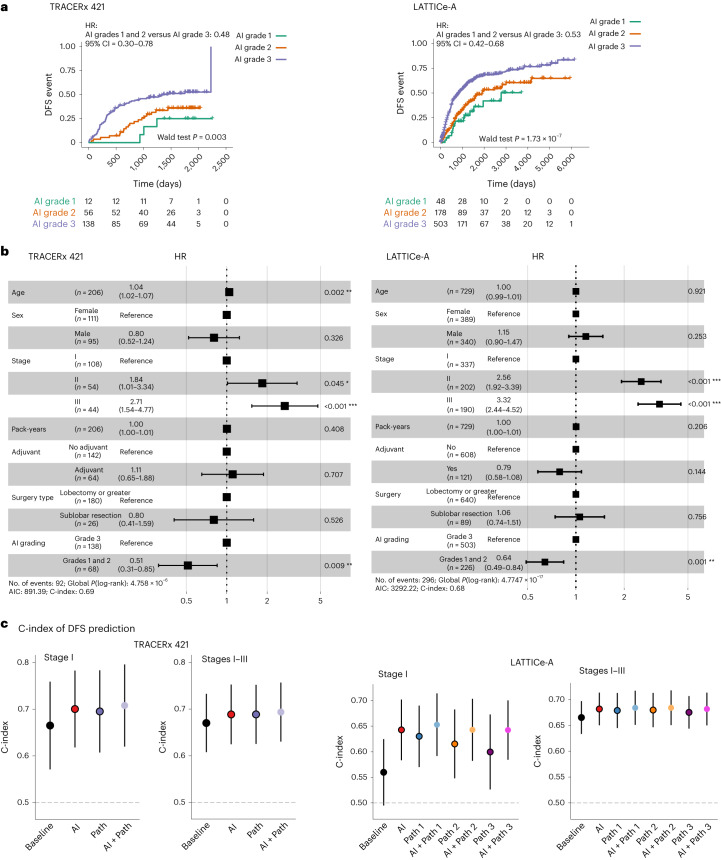
Survival analyses of AI and pathologist grading. **a**, Kaplan–Meier curves illustrating the difference in DFS according to AI grading. **b**, Multivariable Cox regression analyses showing that the prognostic effect of AI grading is independent of age, sex, tumor stage, smoking pack-years, adjuvant therapy and type of surgery (TRACERx 421: *P* = 0.009408, LATTICe-A: *P* = 0.00118). HRs of each variable with 95% CIs are shown on the horizontal axis; the *P* value was derived using a Wald test. *P < 0.05, **P < 0.01, ***P < 0.001. **c**, Comparison of DFS prediction measured according to C-index for stage I (TRACERx 421, *n* = 108; LATTICe-A, *n* = 337) and stage I–III (TRACERx 421, *n* = 206, LATTICe-A, *n* = 729) tumors, where the baseline characteristics included age, sex and tumor stage; AI included baseline parameters and AI grading; path included baseline parameters and pathologist grading; AI + path included baseline parameters, and AI and pathologist gradings. C-indexes with 95% CIs are shown on the vertical axis. AIC, Akaike information criterion. Source data

**Extended Data Fig. 5:**
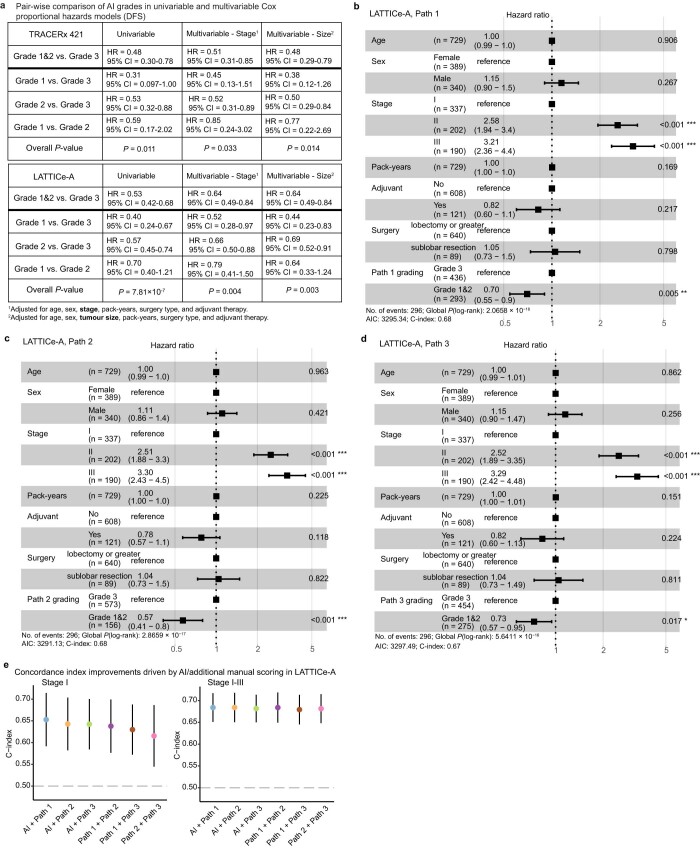
Survival analyses of AI and pathological gradings. **a**. Pair-wise comparison of AI grades in univariable and multivariable Cox proportional hazards models. **b–d**. Multivariable Cox regression analyses showing pathological gradings were independent of age, sex, tumor stage, smoking pack-years, adjuvant therapy, type of surgery in LATTICe-A (P1 = 0.00524, P2 = 0.000913, P3 = 0.0169). HRs of each variable with 95% confidence intervals are shown on the horizontal axis; P value was derived with Wald test. Asterisks indicate: *P < 0.05, **P < 0.01, ***P < 0.001. **e**. Comparison of improvements driven by AI and additional manual scoring for stage I (n = 337) and stage I-III (n = 729) tumors in LATTICe-A, where models included age, sex, tumor stage and gradings from AI or/and pathologists. C-indexes with 95% confidence intervals are shown on the vertical axis. Source data

Patients with IASLC grade 1 and 2 tumors as identified by AI had notably favorable disease-free survival (DFS) compared to patients with IASLC grade 3 tumors in TRACERx 421 (*n* = 206, *P* = 0.003, hazard ratio (HR) = 0.48, 95% confidence interval (CI) = 0.30–0.78) and LATTICe-A (*n* = 729, *P* = 1.73 × 10^−7^, HR = 0.53, 95% CI = 0.42–0.68; Fig. 3a). This prognostic effect remained notable when AI grading was incorporated in a multivariable model (TRACERx 421, *n* = 206, *P* = 0.009, HR = 0.51, 95% CI = 0.31–0.85; LATTICe-A, *n* = 729, *P* = 0.001, HR = 0.64, 95% CI = 0.49–0.84; Fig. 3b). The prognostic effect was slightly changed when tumor stage was replaced by tumor size (TRACERx 421, *P* = 0.004, HR = 0.48, 95% CI = 0.29–0.79; LATTICe-A, *P* = 0.001, HR = 0.64, 95% CI = 0.49–0.84; Extended Data Fig. 5a). The overall prognostic effect of the pair-wise comparison was consistently retained in the univariable (TRACERx 421, *P* = 0.011; LATTICe-A, *P* = 7.81 × 10^−7^) and multivariable analyses (TRACERx 421, tumor stage: *P* = 0.033, tumor size: *P* = 0.014; LATTICe-A, tumor stage: *P* = 0.004, tumor size: *P* = 0.003; Extended Data Fig. 5a).

To determine the prognostic information provided by AI compared to manual scoring and the clinical baseline characteristics, we focused on the large LATTICe-A cohort. While manual IASLC grading from all three pathologists was prognostic (Extended Data Fig. 5b–d), AI grading achieved a comparable performance with all three pathologists (Fig. 3b) in LATTICe-A. When Cox regression models were considered for predicting DFS (baseline; age, sex, tumor stage), AI grading (baseline + automated IASLC grading) and manual grading (baseline + a pathologist’s manual IASLC grading), AI grading achieved a comparable performance with pathologists and clinical baseline for stage I–III tumors in LATTICe-A (*n* = 729, concordance index (C-index): AI = 0.682, 95% CI = 0.650–0.713; path 1 = 0.679, 95% CI = 0.645–0.713; path 2 = 0.680, 95% CI = 0.647–0.713; path 3 = 0.675, 95% CI = 0.644–0.707; baseline = 0.665, 95% CI = 0.633–0.697; Fig. 3c). Consistent performance was observed for stage I–III tumors in TRACERx 421 (*n* = 206, C-index: AI = 0.689, 95% CI = 0.625–0.752; path = 0.689, 95% CI = 0.625–0.752; baseline = 0.670, 95% CI = 0.608–0.733; Fig. 3c). In patients with early-stage tumors, the C-index of AI grading was comparable with pathologist grading but higher than baseline in TRACERx 421 (*n* = 108, C-index: AI = 0.700, 95% CI = 0.618–0.783; path = 0.695, 95% CI = 0.607–0.783; baseline = 0.665, 95% CI = 0.571–0.759; Fig. 3c). However, in LATTICe-A, the association between DFS and AI grading was consistently higher than the grading from pathologists (*n* = 337, C-index: AI = 0.643, 95% CI = 0.584–0.702; path 1 = 0.630, 95% CI = 0.570–0.690; path 2 = 0.615, 95% CI = 0.548–0.683; path 3 = 0.600, 95% CI = 0.526–0.673; baseline = 0.560, 95% CI = 0.495–0.625; Fig. 3c). Furthermore, once AI grading was added to manual grading (Supplementary Table 4), the prognostic value of the combined grading was consistently improved for stage I tumors (increment in C-index for path in TRACERx 421 = 0.013; path 1 = +0.023; path 2 = +0.028; path 3 = +0.043 in LATTICe-A; Fig. 3c), which was marginally higher than adding an additional manual grading in LATTICe-A (Extended Data Fig. 5e and Supplementary Table 5).

Taken together, these data suggest that AI grading adds independent prognostic value for patient stratification, particularly for stage I disease in which clinical decision-making regarding adjuvant therapy following surgery can be challenging in the absence of evidence for outcome benefit.

### Assisting pathologists in challenging scenarios

To evaluate the utility of our AI method to assist pathologists with LUAD grading, we identified four specific scenarios and used the large LATTICe-A cohort with manual grading available from three pathologists. We focused on stage I LUAD tumors, a group of patients with an unmet need for predicting which patients are likely to relapse to guide early intervention, potentially with adjuvant therapy^21^.

**Fig. 4:**
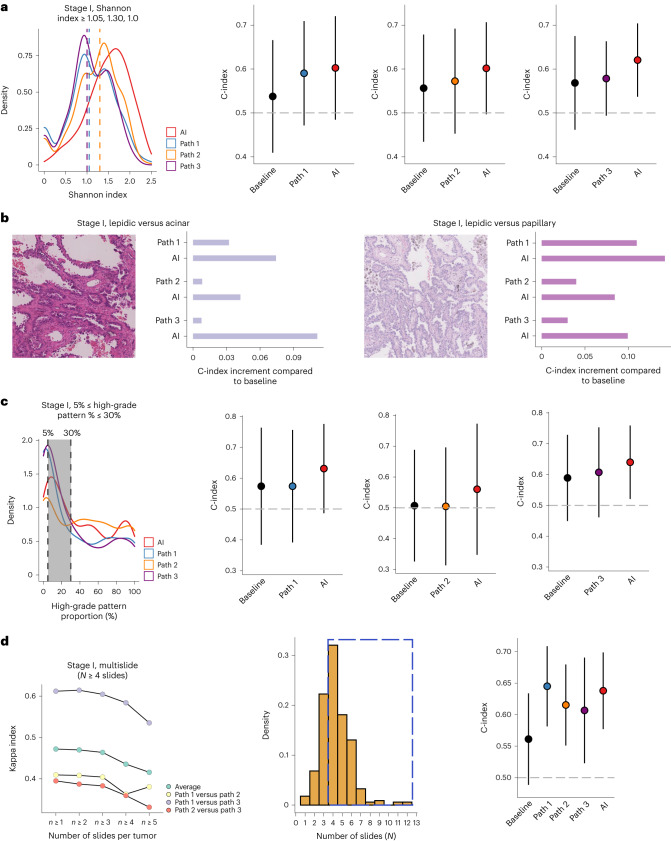
Assistance of AI in challenging scenarios for grading stage I tumors in LATTICe-A. **a**, Scenario 1: tumors with highly diversified growth patterns indicated by the Shannon diversity index (equal to or greater than the median). The vertical dashed lines indicate median values. Comparison of DFS prediction measured using the C-index (*n*1 = 169, *n*2 = 162, *n*3 = 167), where baseline included age and sex, AI included baseline parameters and AI grading, and path included baseline parameters and pathologist grading. C-indexes with 95% CIs are shown on the vertical axis (same with **c**,**d**). **b**, Scenario 2: differentiation between lepidic-predominant and acinar-predominant tumors (*n*1 = 146, *n*2 = 136, *n*3 = 175), and between lepidic-predominant and papillary-predominant tumors (*n*1 = 92, *n*2 = 77, *n*3 = 79). C-index improvement compared with baseline regarding DFS prediction. **c**, Scenario 3: tumors with high-grade patterns between 5% and 30% (*n*1 = 79, *n*2 = 63, *n*3 = 128, gray areas between two vertical dashed lines). **d**, Scenario 4: tumors with no fewer than four slides (*n* = 233, dashed box), for which the interobserver kappa index decreased. Source data

**Extended Data Fig. 6:**
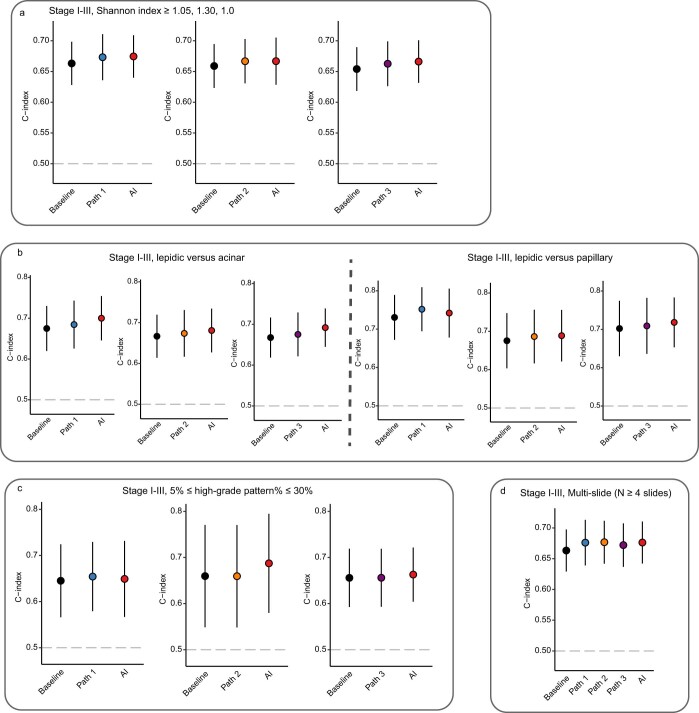
Assistance of AI in grading challenging scenarios for stage I-III tumors in LATTICe-A. **a**. Challenging scenario 1, tumors with highly diversified growth patterns indicated by the Shannon diversity index (n1 = 363, n2 = 361, n3 = 390). **b**. Challenging scenario 2, differentiation between lepidic- and acinar-predominant tumors (n1 = 274, n2 = 222, n3 = 340), and between lepidic- and papillary-predominant tumors (n1 = 162, n2 = 134, n3 = 137). **c**. Challenging scenario 3, tumors with high-grade patterns between 5% and 30% (n1 = 162, n2 = 117, n3 = 252). **d**. Challenging scenario 4, tumors with no less than 4 slides (n = 551). C-indexes of each variable with 95% confidence intervals are shown on the vertical axis. Source data

The first scenario consisted of cases with highly diversified growth patterns indicated by the Shannon diversity index (Fig. 4a), which was notably higher in cases with discrepant predominant patterns between AI and pathologists (Fig. 2d). When evaluated in cases with high growth pattern diversity based on the Shannon index derived from manual scoring, AI grading consistently obtained a higher C-index than pathological grading for DFS prediction (AI = 0.602, 95% CI = 0.485–0.720; path 1 = 0.590, 95% CI = 0.472–0.709, *n*1 = 169; AI = 0.602, 95% CI = 0.497–0.706; path 2 = 0.572, 95% CI = 0.453–0.692, *n*2 = 162; AI = 0.620, 95% CI = 0.537–0.704; path 3 = 0.578, 95% CI = 0.494–0.663, *n*3 = 167; stage I, Fig. 4a; stages I–III, Extended Data Fig. 6a; all models included baseline clinical parameters, same hereafter).

Second, we focused on tumors scored predominantly as lepidic or acinar by each pathologist, excluding any morphologically homogeneous tumor that received a score of 90% or more for either pattern^22^. There is an ongoing difficulty in the histopathological discrimination between in situ and invasive disease^4^, and the distinction between invasive acinar and lepidic growth altered by interstitial fibrosis or iatrogenic compression with alveolar collapse can be particularly difficult. Differences in classification between pathologists can generate a shift between low and medium grade, which was observed among pathologists in the LATTICe-A cohort (Extended Data Fig. 4a). Therefore, these heterogeneously scored lepidic-predominant or acinar-predominant tumors present a challenging scenario to further test the added benefit of an AI grading system. AI grading consistently achieved a better performance in predicting DFS against pathological grading (AI = 0.658, 95% CI = 0.546–0.770; path 1 = 0.616, 95% CI = 0.513–0.718, *n*1 = 146; AI = 0.621, 95% CI = 0.530–0.711; path 2 = 0.587, 95% CI = 0.478–0.695, *n*2 = 136; AI = 0.703, 95% CI = 0.625–0.781; path 3 = 0.599, 95% CI = 0.512–0.687, *n*3 = 175; stage I, Fig. 4b; stages I–III, Extended Data Fig. 6b). There was a similar challenge in distinguishing between lepidic and papillary growth. When predominantly but heterogeneously presented (<90%) lepidic and papillary tumors were investigated in the context of comparing DFS prediction, AI grading consistently achieved a higher C-index (AI = 0.651, 95% CI = 0.420–0.882; path 1 = 0.619, 95% CI = 0.427–0.811, *n*1 = 92; AI = 0.658, 95% CI = 0.449–0.8670; path 2 = 0.614, 95% CI = 0.442–0.786, *n*2 = 77; AI = 0.602, 95% CI = 0.423–0.780; path 3 = 0.532, 95% CI = 0.373–0.692, *n*3 = 79; stage I, Fig. 4b; stages I–III, Extended Data Fig. 6b). The absence of statistical significance could be attributed to the relatively smaller number of patients and events in each group.

The third scenario was the detection of aggressive, high-grade patterns. Although there was a high concordance rate for cases composed predominantly of high-grade patterns (Extended Data Fig. 4e), the proposed IASLC grading system sets a 20% cutoff for high-grade patterns to qualify as grade 3, adding challenges to identify high-grade patterns from non-high-grade pattern-predominant tumors. Therefore, we selected tumors with high-grade patterns (≥5%) at lower abundance (≤30%) as scored by each pathologist and compared their manual grading with AI grading. Such analyses allowed us to examine manually scored tumors, which may be ‘close calls’ among observers when determining the high-grade pattern cutoff. A higher C-index for AI grading was consistently observed compared with all pathologists’ grading in predicting DFS (AI = 0.631, 95% CI = 0.486–0.776; path 1 = 0.574, 95% CI = 0.392–0.757, *n*1 = 79; AI = 0.560, 95% CI = 0.347–0.773; path 2 = 0.505, 95% CI = 0.313–0.696, *n*2 = 63; AI = 0.640, 95% CI = 0.521–0.759; path 3 = 0.607, 95% CI = 0.461–0.753, *n*3 = 128; stage I, Fig. 4c; stages I–III, Extended Data Fig. 6c).

Finally, we considered cases with high numbers of diagnostic slides per tumor (Fig. 4d), defined as four or more slides (*n* = 233, decreased kappa index in Fig. 4d). In these cases, AI grading achieved a C-index higher than average for the manual grading but lower than pathologist 1 in predicting DFS (AI = 0.638, 95% CI = 0.577–0.699; path 1 = 0.645, 95% CI = 0.581–0.709; path 2 = 0.615, 95% CI = 0.551–0.680; path 3 = 0.607, 95% CI = 0.523–0.691; stage I, Fig. 4d; stages I–III, Extended Data Fig. 6d).

These data indicated that our proposed AI method was not inferior to pathological grading and could assist pathologists to grade growth patterns in certain challenging scenarios.

### Acinar morphology and spatial heterogeneity

**Fig. 5:**
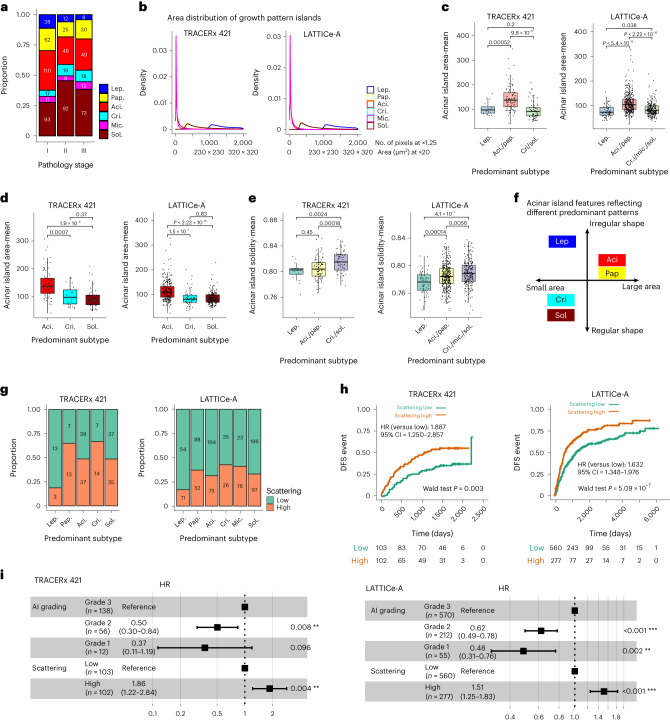
Characterization of tumors with acinar morphological features and spatial heterogeneity. **a**, LUAD subtype distribution across stages in LATTICe-A showing that acinar is the most prevalent pattern in stage I tumors. **b**, Area distribution of growth pattern islands delineated by AI in TRACERx 421 and LATTICe-A, indicating that the areas of acinar islands are similar to micropapillary islands, but smaller than lepidic, papillary, cribriform and solid islands. **c**, Smaller acinar island areas were enriched in lepidic-predominant (TRACERx 421, *P* = 0.0005161, *n* = 108; LATTICe-A, *P* = 5.413 × 10^−12^, *n* = 420) and high-grade-predominant tumors (TRACERx 421, *P* = 9.797 × 10^−11^, *n* = 157; LATTICe-A, *P* < 2.2 × 10^−16^, *n* = 593) compared to acinar-predominant and papillary-predominant tumors. **d**, Acinar island areas were notably smaller in cribriform-predominant tumors compared to acinar-predominant tumors (TRACERx 421, *P* = 0.0006956, *n* = 95; LATTICe-A, *P* = 1.515 × 10^−7^, *n* = 290). **e**, Acinar island shapes were notably regular in high-grade-predominant tumors compared to lepidic-predominant tumors (TRACERx 421, *P* = 0.002439, *n* = 81; LATTICe-A, *P* = 4.118 × 10^−7^, *n* = 295). **c**–**e**, Each point is a tumor; the *y* axis is the mean area (**c**,**d**) or solidity index (**e**) of all the individual acinar islands within a tumor. The *P* value was calculated using a two-sided Wilcoxon rank-sum test not adjusted for multiple comparisons. The median value is indicated by a thick horizontal line; the first and third quartiles are represented by the box edges; the whiskers indicate 1.5× the interquartile range. **f**, Acinar morphological features reflecting different growth patterns; small-area acinar islands with irregular shapes were more likely observed in lepidic-predominant tumors, whereas in cribriform-predominant and solid-predominant tumors, small-area acinar islands with a regular shape were enriched. **g**, Spatial arrangement of acinar islands across predominant subtypes. **h**, Kaplan–Meier curves comparing tumors with low and high levels of acinar scattering for TRACERx 421 and LATTICe-A. **i**, Multivariable Cox regression analyses showing that tumors exhibiting a high degree of acinar scattering were linked to decreased DFS compared to tumors with low acinar scattering, independent of AI grading in TRACERx 421 (*P* = 0.004209) and LATTICe-A (*P* = 2.61 × 10^−5^). HRs of each variable with 95% CIs are shown on the horizontal axis; the *P* value was derived using a Wald test. **P < 0.01, ***P < 0.001. Source data

Precise spatial delineations of growth patterns allowed us to study the spatial configuration of tumors as morphologically distinct pattern islands (Fig. 2a and Extended Data Figs. 2a,b and 3a). Acinar growth, often considered as an intermediate state during the transition of morphological patterns^6,23^, was also the most prevalent pattern in stage I tumors in the LATTICe-A cohort (Fig. 5a). The area of individual acinar islands was similar to that of micropapillary islands, and smaller than those of other patterns (Fig. 5b). These data led us to investigate the importance of morphological features and spatial distribution of acinar islands that may be indicative of histology pattern transition.

**Extended Data Fig. 7:**
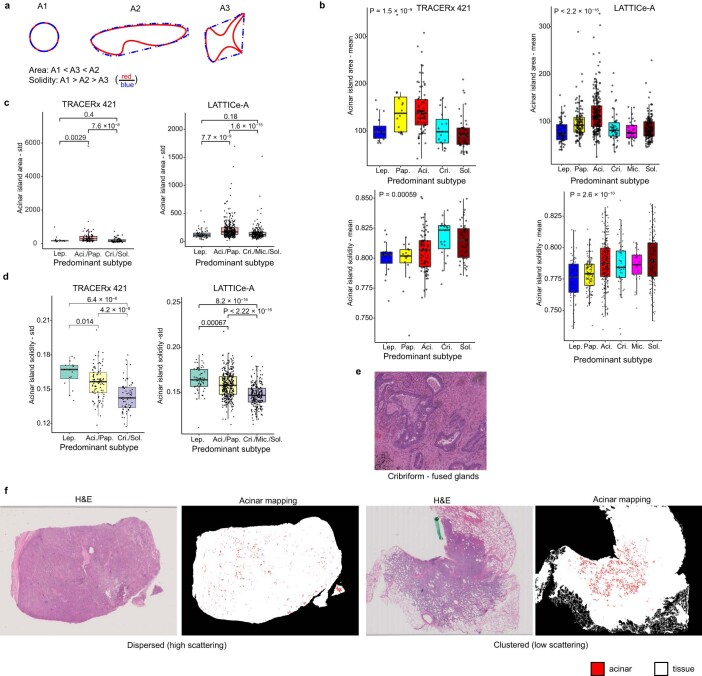
Morphological and spatial analyses of acinar island. **a**. Acinar morphological feature measures, area and solidity index. **b**. Acinar islands are morphologically different among tumors with different predominant patterns (TRACERx 421, P = 1.493 × 10^−9^ and P = 0.0005932, n = 173; LATTICe-A, P < 2.22 × 10^−16^ and P = 2.626 × 10^−10^, n = 654). P value was calculated using a one-way Kruskal-Wallis rank-sum test and not adjusted for the multiple comparisons. **c**. Acinar island areas were less varied in lepidic-predominant (TRACERx 421, P = 0.002889, n = 108; LATTICe-A, P = 7.743 × 10^−9^, n = 420) and high-grade-predominant (TRACERx 421, P = 7.617 × 10^−8^, n = 157; LATTICe-A, P = 1.611 × 10^−15^, n = 593) tumors than acinar- and papillary-predominant tumors. **d**. Acinar island shapes were less varied in high-grade-predominant tumors than lepidic predominant tumors (TRACERx 421, P = 6.374 × 10^−6^, n = 81; LATTICe-A, P = 8.184 × 10^−16^, n = 295). **b**-**d**. Each point is a tumor, y axis is the standard deviation of the area or solidity index for all the individual acinar islands within a tumor. The median value is indicated by a thick horizontal line; the first and third quartiles are represented by box edges; whiskers indicate 1.5 times interquartile range. **c**-**d**. P value was calculated using a two-sided Wilcoxon rank-sum test and not adjusted for the multiple comparisons. **e**. Example illustrating the transition from acinar to cribriform. **f**. Examples of high and low acinar scattering inferred from H&E images with the AI method, deposited in 10.6084/m9.figshare.24599796. Source data

We used area and shape measured using pixel number and solidity index (Extended Data Fig. 7a) to represent the morphological features of individual acinar islands. Acinar island area and shape were notably different in tumors (≥5% of acinar) with different predominant patterns (TRACERx 421 *n* = 173; LATTICe-A *n* = 654; Extended Data Fig. 7b). Smaller acinar islands were enriched in lepidic-predominant tumors compared to acinar-predominant and papillary-predominant tumors (TRACERx 421 *P* = 0.00052; LATTICe-A *P* = 5.4 × 10^−12^; Fig. 5c and Extended Data Fig. 7c). This may reflect the acinar structures in lepidic-predominant disease frequently representing airspaces with iatrogenic collapse^24^. The area of acinar islands in high-grade pattern-predominant (cribriform, micropapillary and solid) tumors were also smaller than those in acinar-predominant and papillary-predominant tumors (TRACERx 421 *P* = 9.8 × 10^−11^; LATTICe-A *P* < 2.22 × 10^−16^; Fig. 5c and Extended Data Fig. 7c). Notably, this area feature was a strong discriminator between acinar-predominant and cribriform-predominant tumors (TRACERx 421 *P* = 0.0007; LATTICe-A *P* = 1.5 × 10^−7^; Fig. 5d), indicating that acini may form differently in acinar-predominant tumors compared to others. The transition from an acinar to a cribriform pattern may frequently occur to large acinar islands through gland fusion (Extended Data Fig. 7e), while smaller acinar structures may remain. Alveolar architectures in airspace detected in acinar-predominant tumors might also be supporting large ‘glands’. Acinar islands with regular shapes were enriched in high-grade-predominant tumors compared with lepidic subtypes (TRACERx 421 *P* = 0.0024; LATTICe-A *P* = 4.1 × 10^−7^; Fig. 5e and Extended Data Fig. 7d), which is again consistent with morphological variance due to the compressibility of lepidic growth. Taken together, the morphological features of acinar islands vary notably in tumors predominantly enriched with different patterns (Fig. 5f).

To investigate the spatial arrangement of acinar patterns, we developed an acinar scattering score that measured the degree of acinus dispersion. A low score indicated locally clustered acinar islands, while a high score implied a dispersion of acinar islands throughout the tissue (Extended Data Fig. 7f). Low acinar scattering was found more frequently in lepidic-predominant tumors compared to all others (TRACERx 421 *P* = 0.017; LATTICe-A *P* = 0.004; Fig. 5g), indicating that clustered acinar islands may reflect the compression induced by iatrogenic collapse and may also suggest that the transition from lepidic to acinar occurs in an organized manner^25^. We next explored acinar scattering in the context of outcome prediction. Tumors with highly scattered acini were associated with reduced DFS compared to lowly scattered tumors (TRACERx 421 *n* = 205, *P* = 0.003, HR = 1.89, 95% CI = 1.25–2.86; LATTICe-A *n* = 837, *P* = 5.09 × 10^−7^, HR = 1.63, 95% CI = 1.35–1.98; Fig. 5h) in univariate analysis. In a multivariable model incorporating acinar scattering and AI grading, acinar scattering was independent of AI grading (TRACERx 421 *P* = 0.004; LATTICe-A *P* = 2.61 × 10^−5^; Fig. 5i). These data suggest that acinar scattering may be a potential pattern reflecting histological transition events, and that high scattering may be a morphological phenotype indicating poor prognosis, which can be assessed from H&E images.

## Discussion

We have developed an AI method ANORAK for the precise classification of growth patterns in LUAD. To the best of our knowledge, this is the first AI method to dissect LUAD growth patterns at the pixel level and be tested in over 1,000 cases, setting a benchmark in automated grading of LUAD. Our method can automatically estimate growth pattern proportions and predominant patterns within a tumor, providing an unbiased and automated pipeline for determining IASLC grading in LUAD. Moreover, the precise delineation of growth patterns can provide insights into the heterogeneous landscape of LUAD, which cannot be addressed by patch-wise classification methods.

The AI method was evaluated in four cohorts, comprising a total of 1,372 tumors. The overall agreement of predominant pattern at the tumor level between AI and pathologists across four cohorts was moderate, which is consistent with the inter-pathologist agreement in the LATTICe-A and DHMC cohorts^13^. Similar results were found in previous studies. Boland et al.^3^ reported an agreement of 51.7% between two pathologists for a large cohort of individuals with LUAD (*n* = 534), while Thunnissen et al.^4^ showed good agreement for typical cases and fair agreement for difficult cases by comparing scores from 26 pathologists. In addition, tumors with a discrepant predominant pattern classification between AI and manual scoring were more heterogeneous compared to tumors in agreement. Previous attempts were made to determine how clonal evolution is reflected in growth pattern heterogeneity through the identification of molecular alterations that accompany the transition between growth patterns^6^. This detailed analysis in a small number of tumors found that changes in expression, rather than mutations, accompanied the transition; as such, clear evidence of divergent tumor clones reflected in the growth pattern was not identified. On a larger scale, in the TRACERx study, although without specific focus on sampling to capture divergent growth patterns, there was a tendency for tumors to evolve from low-grade or mid-grade to higher grade growth patterns in individuals with LUAD where an ancestor–descendant relationship could be described based on clonal or subclonal loss of heterozygosity^22^.

The proposed IASLC grading system was originally introduced to improve prognostication using tumor morphology^2^. In our study, AI grading improved the performance of predicting DFS compared to the baseline and pathological grading for stage I tumors, and be comparable for stage I–III tumors. Moreover, the prognostic value of AI grading was independent of clinical parameters in the TRACERx 421 and LATTICe-A cohorts. In typical clinical practice, the colineage of postsurgical recurrence is not definitively confirmed, although data from the TRACERx 421 cohort showed that only two out of 49 cases of clinically classified postsurgical recurrence were of different lineage using whole-exome sequencing^26^. While we acknowledge that these uncommon events limit the ability to predict recurrence from resection specimens, this applies equally to both our method and established practices.

The LATTICe-A cohort, consisting of 845 tumors with scores from three pathologists, allowed a comprehensive investigation of the clinical impact of the AI method and showed its benefit as a morphological biomarker. This benefit was slightly higher than that brought by an additional manual grading for stage I tumors, and was comparable with additional manual grading for stage I–III tumors. Furthermore, analyses of manual scoring demonstrated that tumors with multiple slides and intratumoral morphological heterogeneity were particularly challenging cases. In these cases, AI grading achieved a stronger predictive ability compared to manual grading for stage I tumors. Because stage I patients frequently receive surgical resection without adjuvant therapy, the accurate prediction of recurrence, to better target individual patients for adjuvant therapies, is critical. These data illustrate the clinical utility of our AI method for stage I tumors, which could potentially be used as an alternative or independent variable to manual grading, or be applied specifically to challenging cases.

The AI method enables the spatial profiling of growth patterns at the pixel level, allowing morphological and spatial heterogeneity analyses at the growth pattern island level. This would be unattainable with alternative manual or patch-wise classification methods. We used the area and solidity index to measure acinar island morphology and found that small acinar islands were enriched in lepidic-predominant and high-grade-predominant tumors, while the shape of these small acini in lepidic-predominant tumors was more irregular than high-grade-predominant tumors. This may reflect tumor cell biological and microenvironmental differences regarding the formation of acinar structures within the context of different predominant architectures. Because acinar morphological features were obtained by averaging thousands of acinar islands within a tumor, noise due to island segmentation was mitigated (Supplementary Figs. 1–7). We also developed a metric for measuring the spatial distribution within the tissue space of acinar islands, termed acinar scattering. Low acinar scattering was notably associated with lepidic-predominant tumors compared to others, suggesting that acinar spatial distribution may reflect the transition of growth patterns toward more aggressive behavior. High acinar scattering was correlated to unfavorable outcomes, independent of AI grading.

This study has some limitations. The Dice coefficient of ANORAK is still limited, indicating that error modes exist. Intratumoral and tumor microenvironment heterogeneity may result in variations in growth pattern morphology, making segmentation more challenging, specifically among lepidic, papillary and acinar patterns. Meanwhile, the patching operation during the training and testing stages may limit the field of view, thus losing context information. Stain color shift may also have the potential for misclassification despite the color augmentations and normalizations applied to mitigate this impact. These factors may contribute to local error modes, which, when accumulated, may result in errors at the WSI level. In addition, because the model counted the number of pixels to determine the predominant pattern per tumor, and the area of micropapillary islands was smaller than the papillary structures^27^, the discrepancy between AI and pathologists regarding papillary-predominant and micropapillary-predominant patterns may be considered another error mode. Furthermore, because we only collected histopathology annotations from invasive non-mucinous LUAD as training data, invasive mucinous and preinvasive tumors with distinct morphologies are therefore outside of the scope, which may generate inaccurate results or completely fail if applied to such samples. In addition, we selected a ‘challenging case series’ from the LATTICe-A cohort, because the other cohorts considered in this study had fewer cases satisfying the selection criteria. However, LATTICe-A is not a screening-based cohort. It is therefore crucial to validate the potential clinical benefits of AI grading in further cohorts that include screening-detected tumors. Because there are no other studies reporting the importance of acinar spatial arrangement, further validations and studies of the biological implications of acinar scattering are needed.

In summary, the AI method we developed can automate the predominant growth pattern and IASLC grading for LUAD tumors, achieving a moderate agreement with pathologists; this was validated in four cohorts consisting of 1,372 cases. In the TRACERx 421 and LATTICe-A cohort, AI grading was an independent prognostic indicator and had a stronger prognostic ability than pathological grading alone for stage I tumors in the LATTICe-A cohort. The prognostic performance of AI grading was further underlined in challenging scenarios consisting of cases with multiple slides and greater intratumoral heterogeneity. Furthermore, specific morphological features of tumor acini have the potential to infer different underlying tumor biology, with the spatial heterogeneity of acinar islands reflecting divergent tumor behavior and prognosis.

## Methods

### Study cohorts

TRACERx is a multi-center, prospective study, which began recruitment in April 2014 (https://clinicaltrials.gov/ct2/show/NCT01888601, approved by an independent research ethics committee, ref. no. 13/LO/1546). Formalin-fixed paraffin-embedded and H&E-stained histopathology diagnostic slides were scanned using the NanoZoomer S210 digital slide scanner (catalog no. C13239-01) and NanoZoomer digital pathology system v.3.1.7 (Hamamatsu) at ×40 (0.228 μm per pixel resolution)^28,29^. LATTICe-A is a retrospective series of all consecutively resected primary LUAD tumors at a single UK surgical center between 1998 and 2014. The work was ethically approved by a UK National Health Service research ethics committee (ref. no. 14/EM/1159) and complies with Strengthening the Reporting of Observational Studies in Epidemiology guidelines. All archived slides containing tumor material were used to capture the full diversity of each lesion. Slides were dearchived and scanned using a Hamamatsu NanoZoomer XR at ×40 (0.226 μm per pixel resolution)^23,29^. Available diagnostic slides from the TCGA LUAD^30^ were downloaded from https://portal.gdc.cancer.gov/ in 2021. The DHMC^13^ was downloaded from https://bmirds.github.io/LungCancer/ in 2021. Further information on the research design is available in the Nature Research Reporting Summary linked to this article.

The training set of the AI method consisted of 49 WSIs from 49 patients in the TRACERx 100 cohort^28,29^. The WSIs were sparsely annotated by three independent thoracic subspeciality pathologists, yielding 3,662 patches (768 × 768 pixels at ×20, approximately 0.45 μm per pixel) of annotations for six typical growth patterns (Extended Data Fig. 1a) and non-tumor areas, for example, normal tissue and blank areas.

The AI method was then applied and evaluated on a total of 5,540 WSIs from four cohorts, which were collected, processed and scanned independently. This included patients with invasive non-mucinous LUAD as primary diagnosis (excluding adenocarcinoma in situ, minimally invasive adenocarcinomas and other variants) from the TRACERx 421 cohort (*n* = 206, 1,184 slides)^22,26^, LATTICe-A cohort (*n* = 845, 3,979 slides)^23^, TCGA LUAD cohort (*n* = 178, 234 slides)^30^, DHMC cohort (*n* = 143, 143 slides)^13^ (Table 1). TRACERx 100 is a subset of TRACERx 421. For the TRACERx 421 and LATTICe-A cohorts, slides were from all the diagnostic blocks containing tumor cells. For the DHMC cohort and most patients (91%) in the TCGA cohort, only one slide was available. Hence, we only considered these two cohorts for agreement performance comparison. No statistical method was used to predetermine sample size but our sample sizes are similar to those reported in previous publications^13,22,26,29,30^ and subject to available diagnostic slides. Blinding and randomization were not relevant because this was an observational study. Patients were not allocated to any interventions and they were followed up and assessed as per routine practice. No results from this study were reported back to patients, so there is no likelihood of people changing their behaviors based on these findings. The deep learning model was trained without knowing the outcome of patients, which represents a form of blinding.

Manual pathological grading of growth patterns, as well as individual pattern proportion scoring, were available for the TRACERx 421, LATTICe-A and TCGA cohorts. The DHMC cohort only had predominant pattern data for each slide. In the LATTICe-A cohort, three independent consultant-level thoracic subspeciality pathologists provided growth pattern scoring for each tumor.

In the TRACERx 421 cohort, DFS was defined as the period from the date of registration to the time of radiological confirmation of the recurrence of the primary tumor registered for the TRACERx or the time of death by any cause. During the follow-up, three participants with LUAD (CRUK0512, CRUK0428 and CRUK0511) developed new primary cancer and subsequent recurrence from either the first primary lung cancer or the new primary cancer diagnosed during the follow-up. These cases were censored at the time of the diagnosis of the new primary cancer for DFS analysis because of the uncertainty of the origin of the third tumor^22^.

In the LATTICE-A cohort, recurrence data were obtained from the examination of patient records, notably paper notes and radiological databases, to identify the date of radiologically or biopsy-confirmed recurrence. Cancer-specific death was determined by the presence of lung cancer in the cause of death in the death certificate. Overall survival refers to the date of death.

### Deep learning model architecture

We developed a deep learning-based model^14^ ANORAK which leveraged cross-stream interaction to recognize and segment six histological patterns (lepidic, acinar, papillary, micropapillary, cribriform and solid) on WSIs at the pixel level. The model applied ResNet50 (ref. ^31^) as the backbone with customized modifications to account for the limited training data. It encoded three streams (coarse, intermediate and fine) with different scales of information to gather abundant features at different resolutions (×10 at approximately 0.9 μm per pixel, ×5 and ×2.5). The first-order attention (Extended Data Fig. 1c) introduced global contextual information at an early stage to guide low-level feature learning and enable the first round of interactions between streams. Each output in the coarse and intermediate streams was then fed into a convolution layer to align the depth dimension with the fine stream output. A PPM^15^ (Extended Data Fig. 1c) was used to integrate high-level features. Afterwards, such features were forwarded to a second-order attention module, learning the relationship of streams to extract more discriminative features, and driving high-level feature exchanging between streams (Extended Data Fig. 1c and Fig. 1a).

### Implementation and evaluation

Before training, the annotated tiles were divided into nonoverlapping patches, except for patches at the bottom and right edges, with a size of 768 × 768 pixels at ×20. During training, four data augmentation strategies were used to mitigate overfitting: random rotation within 90 degrees; random width-shift and height-shift up to 20% of the input width and height; randomly zooming in or out in a range of (0.8, 1.2); and random adjustment of the saturation within (0.8, 2.0) and hue within (−0.1, 0.1). Color augmentation was not applied to the cross-validation stage because data were from the same cohort. The model was trained for 60 epochs with a batch size of eight. Cross-entropy loss was applied as the objective function, which was minimized by the Adam optimizer with a step-wise learning rate. The initialization rate was set to 10^−3^ for the first ten epochs; then, it was decreased by ten times for the next 40 epochs, which was then followed by another ten times of decreasing (10^−5^) for the remaining ten epochs. The pipeline was implemented with Python v.3.8, tensorflow-gpu v.2.2, keras v.2.4.3, h5py v.2.10.0, numpy v.1.20.3, opencv-python v.4.5.3.56, pandas v.1.3.2, pillow v.8.3.1 and scipy v.1.7.1.

The ablation experiments at the patch level included comparisons with the baseline method (single-stream), multi-stream with the element-wise add combination (multi-ADD), multi-stream with first-order attention alone (multi-FO), multi-stream with second-order attention alone (multi-SO) and the proposed ANORAK model (multi-FO and multi-SO). The proposed model was compared against other widely used approaches in semantic segmentation, including attention U-Net^17^, DeepLabV3+ (ref. ^18^), DANet^19^ and MedT^20^. We applied the Dice coefficient to evaluate segmentation performance at the patch level and the agreement of predominant patterns to assess prediction at the WSI level. Comparisons were conducted with fivefold cross-validation for the TRACERx 100 cohort (*n* = 53) and on a subset of the LATTICe-A cohort (*n* = 50), an independent dataset to the training dataset.

### Growth pattern and grading inference

Each WSI was divided into tiles of 2,000 × 2,000 pixels with the magnification downsampled to ×20 (approximately 0.45 μm per pixel)^29^. Each tile was then normalized to a target image to align the color before feeding it to the well-trained deep learning model, which, in turn, generated corresponding masks for all growth pattern regions detected at the pixel level. The tile masks were then stitched and further downsampled to ×1.25 (approximately 7.2 μm per pixel). Small components were empirically removed as postprocessing; lepidic patterns that were less than approximately 0.05 mm^2^, and papillary, cribriform and solid patterns that were less than approximately 0.015 mm^2^ were removed.

The predominant pattern and grading were inferred from a stitched and downsampled mask (approximately 7.2 μm per pixel). The growth pattern proportion for each tumor was computed as the proportion across all slides of a given tumor:

$$g_j=\frac{{\sum }_{i=1}^{m}{S}_{ij}}{{\sum }_{i=1}^{m}{\sum }_{j}^{n=6}{S}_{ij}}$$

$${P}={\rm{argmax}}({g}_{j})$$

where *g*_*j*_ is a proportion for the *j* pattern, *j* represents lepidic, acinar, papillary, cribriform, micropapillary and solid, *i* is the *i*-th slide, *m* is the number of slides per tumor, *n* is the number of patterns and *S*_*ij*_ is the number of pixels identified for the *j* pattern with the *i*-th slide. The predominant pattern, *P*, is determined as the pattern with the highest proportion. The growth pattern grading driven by AI followed the IASLC grading system^2^: grade 1, lepidic-predominant tumors with less than 20% of high-grade patterns (solid, micropapillary, cribriform); grade 2, acinar-predominant or papillary-predominant tumors with less than 20% of high-grade patterns; and grade 3, any tumor with 20% or more high-grade patterns.

### Agreement between AI and pathological scores with regard to predominant patterns

The strongest correlation for growth pattern proportion between the AI and manual estimates was observed for the solid pattern (TRACERx 421, rho = 0.79; LATTICe-A correlations against each pathologist’s scoring, rho1 = 0.80, rho2 = 0.77, rho3 = 0.78; TCGA, rho = 0.67; Fig. 2b and Supplementary Table 1), followed by acinar (TRACERx 421, rho = 0.69; LATTICe-A, rho1 = 0.67, rho2 = 0.58, rho3 = 0.65; TCGA, rho = 0.56; Fig. 2b and Supplementary Table 1). A moderate correlation was observed for the micropapillary subtype (TRACERx 421, rho = 0.35; LATTICe-A, rho1 = 0.35, rho2 = 0.42, rho3 = 0.40; TCGA, rho = 0.44; Fig. 2b and Supplementary Table 1). Compared with other patterns, solid-predominant tumors had the highest agreement levels between AI and manual scoring (TRACERx 421, 85.5%; LATTICe-A, 85.4%, 79.9%, 85.3% against three pathologists; TCGA, 72%; DHMC, 90.2%; Fig. 2c). A lower agreement rate was observed for micropapillary-predominant tumors (TRACERx 421, 0%; LATTICe-A, 38.1%, 19.7% and 50% against three pathologists; TCGA, 20%; DHMC, 0%; Fig. 2c). Most discrepant micropapillary-predominant cases were identified as papillary and acinar by AI (TRACERx 421, 40%; LATTICe-A, 42.8%, 63.1%, 43.7%; TCGA, 60%; DHMC, 100%), suggesting that micropapillary islands frequently mixed with acinar or papillary in micropapillary-predominant tumors.

### C-index measuring prognostic ability

We used the C-index to measure the prognostic ability of the survival models. Cox regression models were considered for predicting DFS; specifically, the baseline model included age, sex, tumor stage (excluded for stage I tumors as the stage information remains the same). The AI grading-based model included clinical baseline characteristics and automated IASLC grading. The manual grading-based model included clinical baseline characteristics together with a pathologist’s manual IASLC grading. When excluding clinical parameters, AI grading achieved a comparable C-index with pathological grading in stage I (TRACERx 421: AI = 0.588, 95% CI = 0.483–0.692; path = 0.593, 95% CI = 0.461–0.724; LATTICe-A: AI = 0.616, 95% CI = 0.571–0.661; path 1 = 0.609, 95% CI = 0.563–0.656; path 2 = 0.593, 95% CI = 0.545–0.641; path 3 = 0.571, 95% CI = 0.483–0.658; Supplementary Table 6) and stage I–III tumors (TRACERx 421: AI = 0.588, 95% CI = 0.547–0.630; path = 0.581, 95% CI = 0.530–0.632; LATTICe-A: AI = 0.577, 95% CI = 0.554–0.600; path 1 = 0.577, 95% CI = 0.552–0.603; path 2 = 0.574, 95% CI = 0.551–0.597; path 3 = 0.569, 95% CI = 0.546–0.591; Supplementary Table 6).

### Acinar morphological features

The pixel number and solidity index, that is, the proportion of pixels in the convex hull that were also in a region of interest, were used to measure the individual acinar island area and shape generated by the AI method. A higher solidity index indicated a more regular shape. The average area and solidity index of all the individual acinar islands identified from the available slides were taken as the tumor-level features.

### Acinar scattering score

We adapted an established score, standard distance^32^, to measure the spatial distribution of acinar patterns, which we termed ‘acinar scattering’:

$$d=\sqrt{\frac{{\sum }_{i=1}^{n}{({x}_{i}-{x}_{0})}^{2}+{\sum }_{i=1}^{n}{(\,{y}_{i}-{y}_{0})}^{2}}{n\times N}}$$

where *d* is the standard distance, *n* is the number of isolated acinar islands within the tissue identified by the proposed AI method, *N* is the area of the tissue, (*x*_*i*_, *y*_*i*_) is the centroid of an acinar island and (*x*_0_, *y*_0_) is the mean center of all the acinar islands.

$${x}_{0}=\frac{{\sum }_{i=1}^{n}{x}_{i}}{n},\ {y}_{0}=\frac{{\sum }_{i=1}^{n}{y}_{i}}{n}$$

A higher acinar scattering score indicated a more scattered distribution of acini across the tissue. The median value of all available slides for a given tumor was taken as the tumor-level score. The optimal cutoff (0.36) separating tumors into low-scattering and high-scattering groups was selected from the discovery cohort, LATTICe-A, which was then applied directly to the TRACERx 421 cohort.

**Extended Data Fig. 8:**
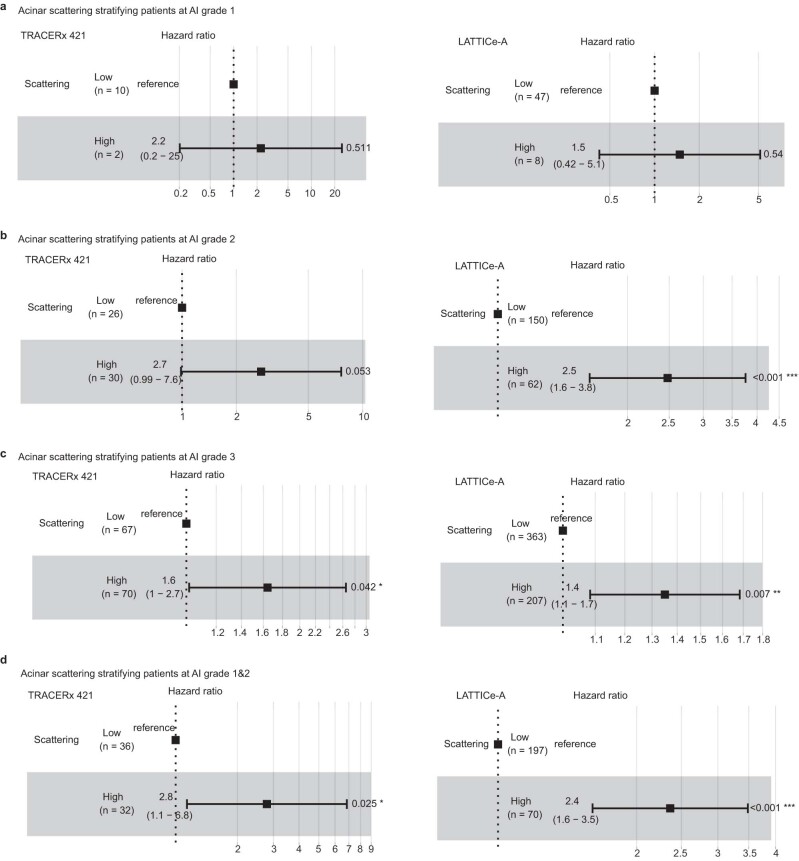
Acinar scattering stratifying subgroups of AI grading. **a**. Acinar scattering stratifying patients at AI grade 1 (TRACERx 421 P = 0.5112, n = 12; LATTICe-A P = 0.5397, n = 55). **b**. Acinar scattering stratifying patients at AI grade 2 (TRACERx 421 P = 0.0533, n = 56; LATTICe-A P = 1.947 × 10^−5^, n = 212). **c**. Acinar scattering stratifying patients at AI grade 3 (TRACERx 421 P = 0.04235, n = 137; LATTICe-A P = 0.007446, n = 570). **d**. Acinar scattering stratifying patients at AI grades 1&2 (TRACERx 421 P = 0.02517, n = 68; LATTICe-A P = 1.387 × 10^−5^, n = 267). HRs of each variable with 95% confidence intervals are shown on the horizontal axis. P value was derived with Wald test, and not adjusted for multiple comparisons. Asterisks indicate: *P < 0.05, **P < 0.01, ***P < 0.001. Source data

In a univariable model, acinar scattering was prognostic of DFS for LATTICe-A in grade 2 and 3 tumors, respectively (grade 2, *n* = 212, *P* = 1.95 × 10^−5^, HR = 2.48, 95% CI = 1.63–3.76; grade 3, *n* = 570, *P* = 0.007, HR = 1.35, 95% CI = 1.08–1.68; Extended Data Fig. 8b,c), but not in grade 1 tumors (Extended Data Fig. 8a). In the TRACERx 421 cohort, high acinar scattering was associated with reduced DFS in grade 3 tumors (*n* = 137, *P* = 0.042, HR = 1.64, 95% CI = 1.01–2.65) and remained borderline in grade 2 tumors (*n* = 56, *P* = 0.053, HR = 2.74, 95% CI = 0.99–7.61), but was not notable in grade 1 tumors. The lack of statistical significance was probably due to the smaller number of patients and events in the grade 1 subgroup. When merging grade 1 and 2 tumors, the prognostic effect of acinar scattering was observed (TRACERx 421, *n* = 68, *P* = 0.025, HR = 2.79, 95% CI = 1.14–6.85; LATTICe-A, *n* = 267, *P* = 1.39 × 10^−5^, HR = 2.36, 95% CI = 1.60–3.48; Extended Data Fig. 8d).

### Statistics and reproducibility

Correlation tests used Spearman’s method and were generated using the function cor.test from the stats v.4.1.2R package. Confusion matrices were obtained using the function confusionMatrix from the caret v.6.0-93R package. Fleiss’ kappa was computed to assess the agreement among observers using the function kappam.fleiss from the irr v.0.84.1R package. Survival analyses were conducted using the Kaplan–Meier estimator (ggsurvplot R function from the survminer v.0.4.9 and survival v.3.2-13R packages) as well as the Cox model (coxph R function, displayed using the ggforest R function). The differences between grade strata Kaplan–Meier curves were determined using Wald tests. Forest plots showed the HR on the *x* axis; each variable’s HR was plotted and annotated with a 95% CI. All HRs were computed for all time points (the whole survival curve was not at a specific time point). For statistical comparisons among groups, a two-sided, nonparametric, unpaired Wilcoxon rank-sum test was used for the continuous variables, while a Fisher’s exact test was used for the categorical variables. A Kruskal–Wallis test was used for comparisons among over two groups, unless stated otherwise. Predictive performance was assessed using a C-index^33^ within 5 years, computed with the function Inf.Cval from the survC1 v.1.0-3R package. Multicollinearity between AI and manual grading, and between two manual gradings were assessed using the function vif from the car v.3.0-12R package. All statistical tests were two-sided and *P* < 0.05 was considered as statistically significant. To adjust *P* values for multiple comparisons, the Benjamini–Hochberg method was used. The packages tidyverse v.2.0.0 and tidyr v.1.3.0 were used for data processing in R. Plotting was done using ggplot2 v.3.4.1, RColorBrewer v.1.1-3 and ggpubr v.0.5.0R packages. All statistical analyses were conducted in R v.4.1.2.

### Reporting summary

Further information on research design is available in the Nature Portfolio Reporting Summary linked to this article.

### Supplementary information

Supplementary Information Supplementary Figs. 1–7.

Reporting Summary

Supplementary Table Supplementary Tables 1–6.

Supplementary Data 1 Source Data for Supplementary Figs. 1–7. Reproducing acinar morphology by using 50% of acinar islands per tumor and repeating ten times.

### Source data

Source Data Figs. 1, 2–5 and Extended Data Figs. 5–8 The source data analyzed in this study are provided in a single file, with named tabs for each cohort. These data are used across Figs. 1b and 2–5, and Extended Data Figs. 5–8.

## Data Availability

The training dataset consisting of annotations on small image tiles have been deposited in Zenodo (10.5281/zenodo.10016027). Previously published image data that were reanalyzed in this study can be requested from https://bmirds.github.io/LungCancer/. The human LUAD diagnostic slide images were derived from the TCGA Research Network at https://portal.gdc.cancer.gov/. Images generated by the AI model in Fig. 2a and Extended Data Figs. 2, 3a and 7f can be accessed at figshare (10.6084/m9.figshare.24599796). For the TRACERx study, all of the scanned diagnostic histological images have a study number label embedded in the file that prevents complete anonymization. Therefore, these images cannot be shared, in line with the ethical approval for the study. Requests for access to the TRACERx dataset for academic noncommercial research purposes can be submitted through the Cancer Research UK and UCL Cancer Trials Centre (ctc.tracerx@ucl.ac.uk) and are subject to review of a project proposal that will be evaluated by a TRACERx data access committee, entering into an appropriate data access agreement and any applicable ethical approvals. The time frame of response to requests is about 6 months. LATTICe-A study data and materials are currently subject to a material and data transfer agreement between the University of Leicester, the University of Cambridge and NHS Greater Glasgow and Clyde, which includes a restricted access period of 5 years, precluding any access by other third parties during this time. After the 5-year period, restricted access data can be accessed by application to NHS Greater Glasgow and Clyde Biorepository (clare.orange@ggc.scot.nhs.uk; john.lequesne@glasgow.ac.uk) as custodians; the data access request will be reviewed and released under their research ethics committee-approved tissue bank protocols. Requests will be reviewed and approved within 6–8 weeks and will be accompanied by a data sharing agreement detailing the conditions and restrictions of use and publication. Source data are provided with this paper.

## Code availability

The AI pipeline for growth pattern segmentation is available at https://github.com/xi11/AIgrading. All code used for the analyses was developed in R v.4.1.2 and is available to reproduce all figures (https://github.com/xi11/AIgrading).
