# Supplementary material for: The artificial intelligence-based model ANORAK improves histopathological grading of lung adenocarcinoma
Source: Nat Cancer. 2024 Jan 10;5(2):347–63. doi: 10.1038/s43018-023-00694-w (PMC10899116; doi:10.1038/s43018-023-00694-w)
Supplement: Supplementary file 1 — Supplementary Figs. 1–7. [file 43018_2023_694_MOESM1_ESM.pdf]

nature cancer

Technical Report https://doi.org/10.1038/s43018-023-00694-w

The artificial intelligence-based model ANORAK improves histopathological grading of lung adenocarcinoma

In the format provided by the authors and unedited

Reproduce Figure 5c

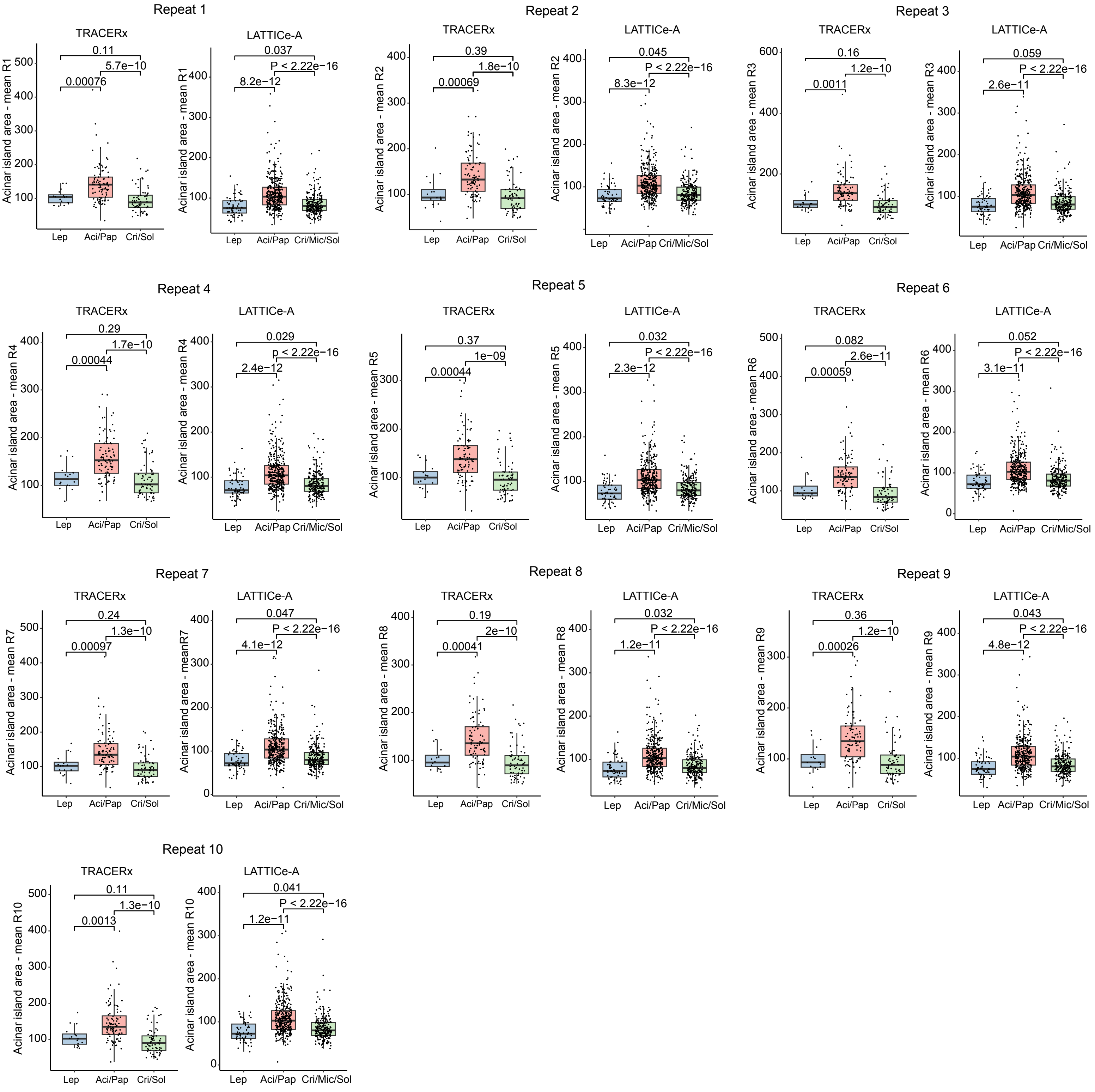

Supplementary Figure 1. Reproducing Figure 5c by using 50% of acinar islands per tumor and repeating 10 times. Similar tendency was observed, smaller acinar island areas were enriched in lepidic-predominant (TRACERx 421, P1=0.0007559035, P2=0.0006877893, P3= 0.0011298166, P4= 0.0004389680, P5=0.0004389680, P6=0.0005868037, P7=0.0009693622, P8=0.0004112280, P9=0.0002583417, P10= 0.0013145254, n=108; LATTICe-A, P1=8.202093e-12, P2=8.267623e-12, P3=2.606164e-11, P4=2.430580e-12, P5=2.295707e-12, P6=3.139325e-11, P7=4.084449e-12, P8=1.248321e-11, P9=4.837327e-12, P10=1.162686e-11, n=420) and high-grade-predominant tumors (TRACERx 421, P1=5.674948e-10, P2=1.801276e-10, P3=1.210760e-10, P4=1.679765e-10, P5=9.956295e-10, P6=2.627624e-11, P7=1.299022e-10, P8=1.976729e-10, P9=1.239508e-10, P10=1.329817e-10, n=157, LATTICe-A, P1-P10<2.22e-16, n=593) compared to acinar- and papillary predominant tumors. P value was calculated using a two-sided Wilcoxon rank-sum test and not adjusted for the multiple comparisons. The median value is indicated by a thick horizontal line; the first and third quartiles are represented by box edges; whiskers indicate 1.5 times interquartile range

Reproduce Figure 5d

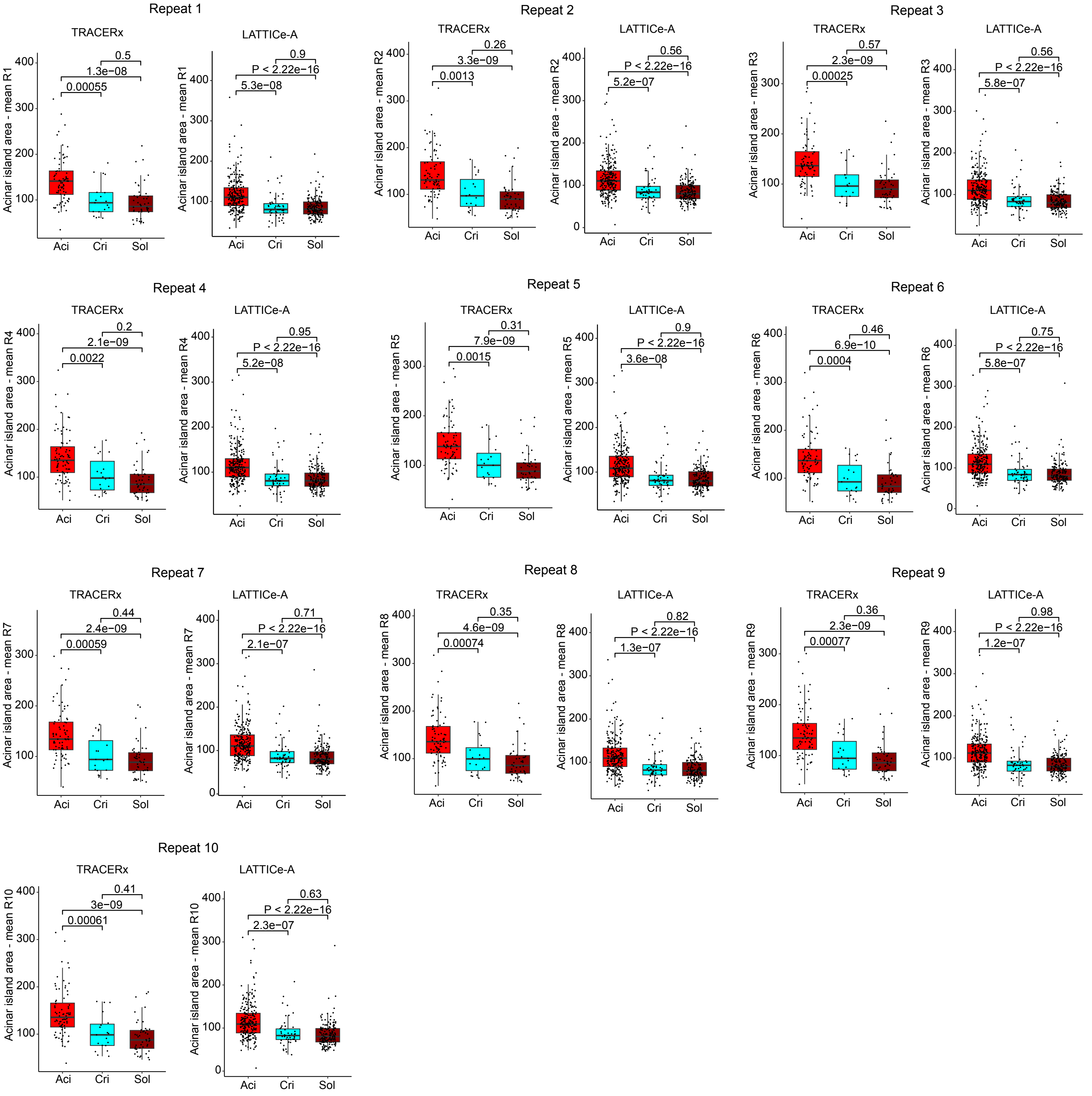

Supplementary Figure 2. Reproducing Figure 5d by using 50% of acinar islands per tumor and repeating 10 times. Similar tendency was observed, acinar island areas were significantly smaller in cribriform-predominant tumors compared to acinar-predominant tumors (TRACERx 421, P1=0.0005473416, P2=0.0013065160, P3=0.0002510971, P4=0.0022403011, P5=0.0015347861, P6= 0.0003998452, P7=0.0005863708, P8=0.0007443142, P9=0.0007698615, P10=0.0006068427, n=95; LATTICe-A, P1=5.277080e-08, P2=5.242959e-07, P3=5.828643e-07, P4=5.222431e-08, P5=3.582194e-08, P6=5.828643e-07, P7=2.086572e-07, P8=1.341967e-07, P9=1.152795e-07, P10=2.281907e-07, n=290). P value was calculated using a two-sided Wilcoxon rank-sum test and not adjusted for the multiple comparisons. The median value is indicated by a thick horizontal line; the first and third quartiles are represented by box edges; whiskers indicate 1.5 times interquartile range.

Reproduce Figure 5e

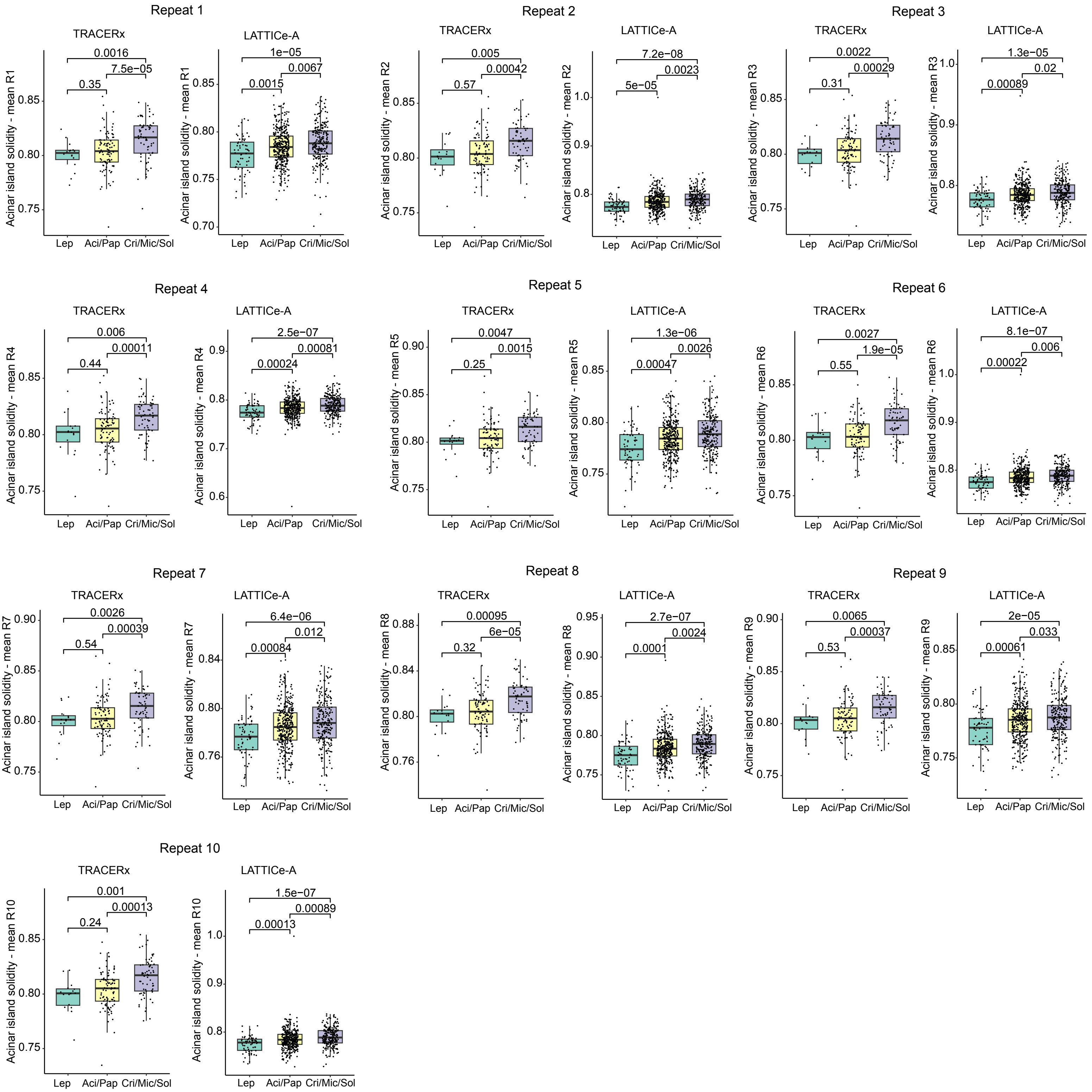

Supplementary Figure 3. Reproducing Figure 5e by using 50% of acinar islands per tumor and repeating 10 times. Similar tendency was observed, acinar island shapes were significantly regular in high-grade-among tumors compared to lepidic-predominant tumors (TRACERx 421, P1=0.0015707241, P2=0.0050250768, P3=0.0021665957, P4=0.0060306432, P5=0.0046672354, P6=0.0027424893, P7= 0.0026377028, P8=0.0009543957, P9=0.0064812373, P10= 0.0010383955, n=81; LATTICe-A, P1=1.004164e-05, P2=7.231972e-08, P3=1.306729e-05, P4=2.454135e-07, P5=1.314198e-06, P6=8.070471e-07, P7=6.411760e-06, P8=2.733602e-07, P9=1.972597e-05, P10=1.489452e-07, n=295). P value was calculated using a two-sided Wilcoxon rank-sum test and not adjusted for the multiple comparisons. The median value is indicated by a thick horizontal line; the first and third quartiles are represented by box edges; whiskers indicate 1.5 times interquartile range.

Reproduce Extended Data Figure 7b - TRACERx 421

Acinar island area

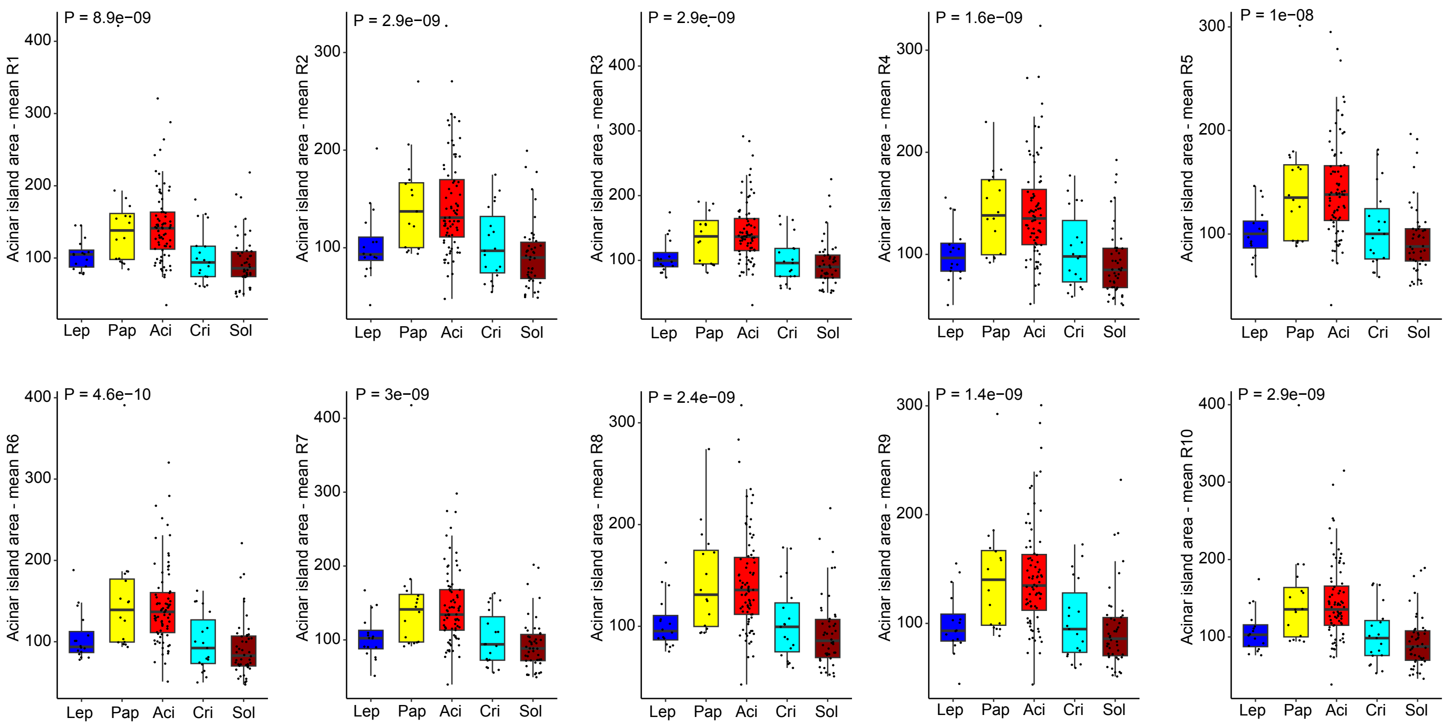

Acinar island solidity

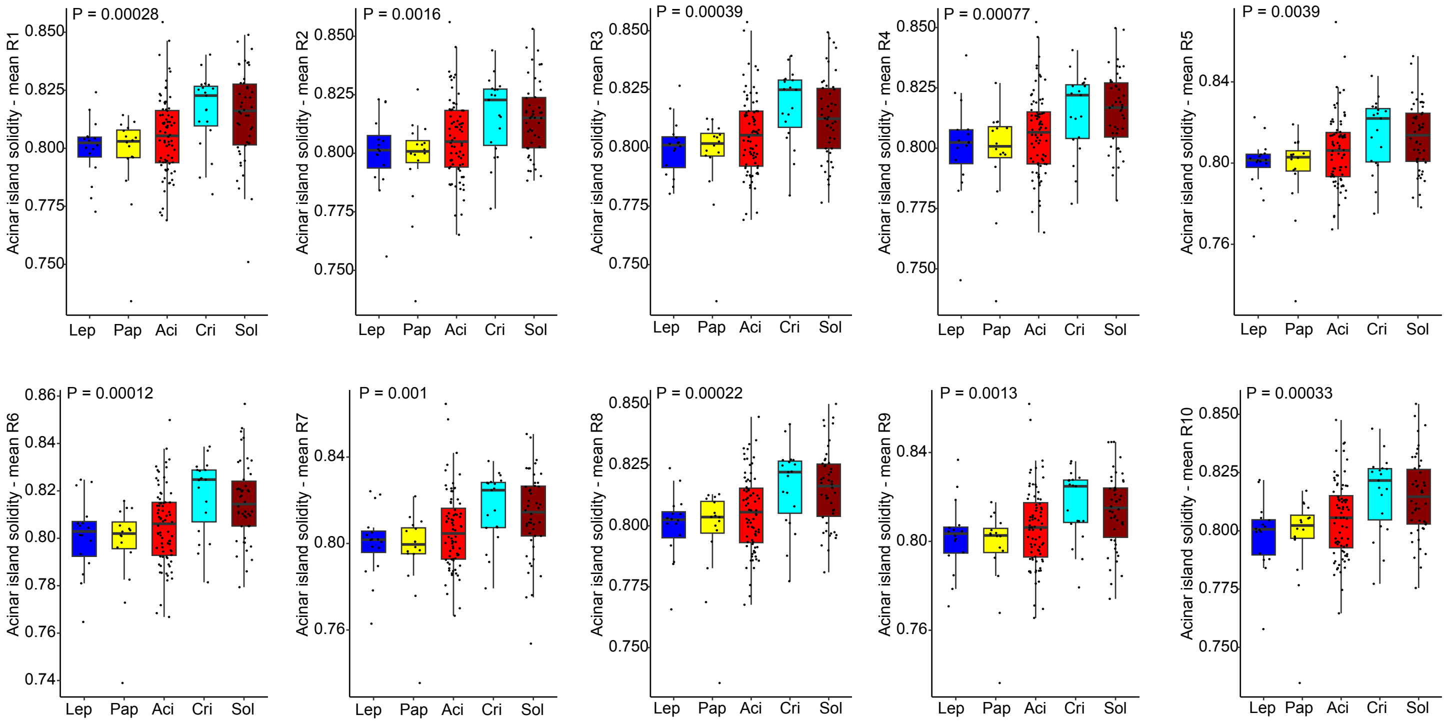

Supplementary Figure 4. Reproducing Extended Data Figure 7b by using 50% of acinar islands per tumor and repeating 10 times for TRACERx 421 cohort. Similar tendency was observed, acinar islands are morphologically different among tumors with different predominant patterns (acinar area: P1=8.858616e-09, P2=2.912074e-09, P3= 2.914101e-09, P4=1.604693e-09, P5=1.040075e-08, P6=4.568240e-10, P7=2.954117e-09, P8=2.419632e-09, P9=1.432690e-09, P10=2.870097e-09; acinar shape: P1=0.0002813199, P2=0.0016378670, P3=0.0003854758, P4=0.0007675337, P5= 0.0038669754, P6=0.0001224812, P7=0.0010425651, P8=0.0002189155, P9= 0.0012869787, P10=0.0003278952, n=173). P value was calculated using a one-way Kruskal-Wallis rank-sum test and not adjusted for the multiple comparisons. The median value is indicated by a thick horizontal line; the first and third quartiles are represented by box edges; whiskers indicate 1.5 times interquartile range.

Reproduce Extended Data Figure 7b - LATTICe-A

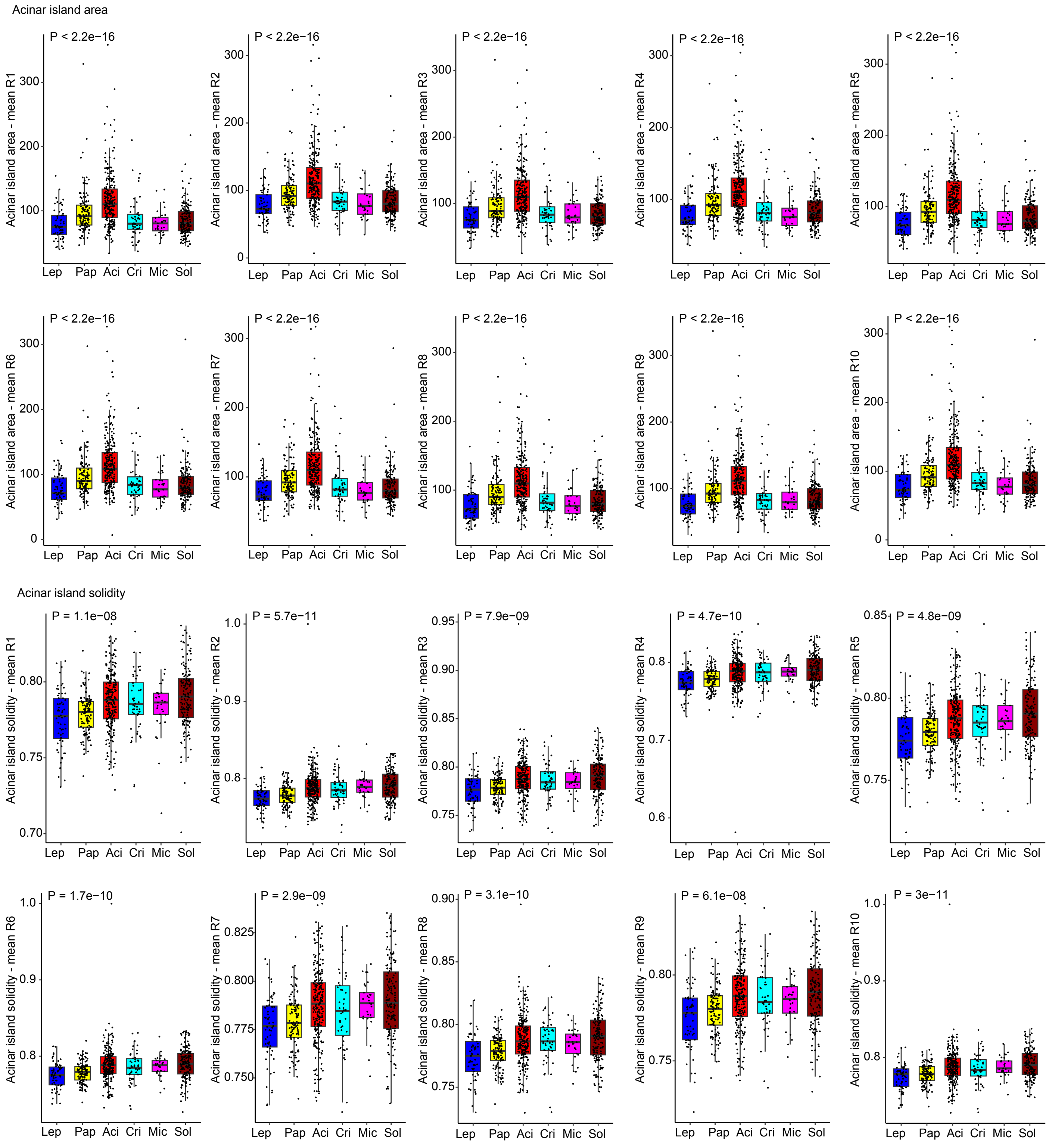

Supplementary Figure 5. Reproducing Extended Data Figure 7b by using 50% of acinar islands per tumor and repeating 10 times for LATTICe-A cohort. Similar tendency was observed, acinar islands are morphologically different among tumors with different predominant patterns (acinar area: P1-P10<2.22e-16; acinar shape: P1=1.087018e-08, P2=5.718833e-11, P3=7.863343e-09, P4=4.702437e-10, P5=4.823926e-09, P6=1.664403e-10, P7=2.940408e-09, P8=3.137030e-10, P9= 6.110131e-08, P10=2.951022e-11, n=654). P value was calculated using a one-way Kruskal-Wallis rank-sum test and not adjusted for the multiple comparisons. The median value is indicated by a thick horizontal line; the first and third quartiles are represented by box edges; whiskers indicate 1.5 times interquartile range.

Reproduce Extended Data Figure 7c

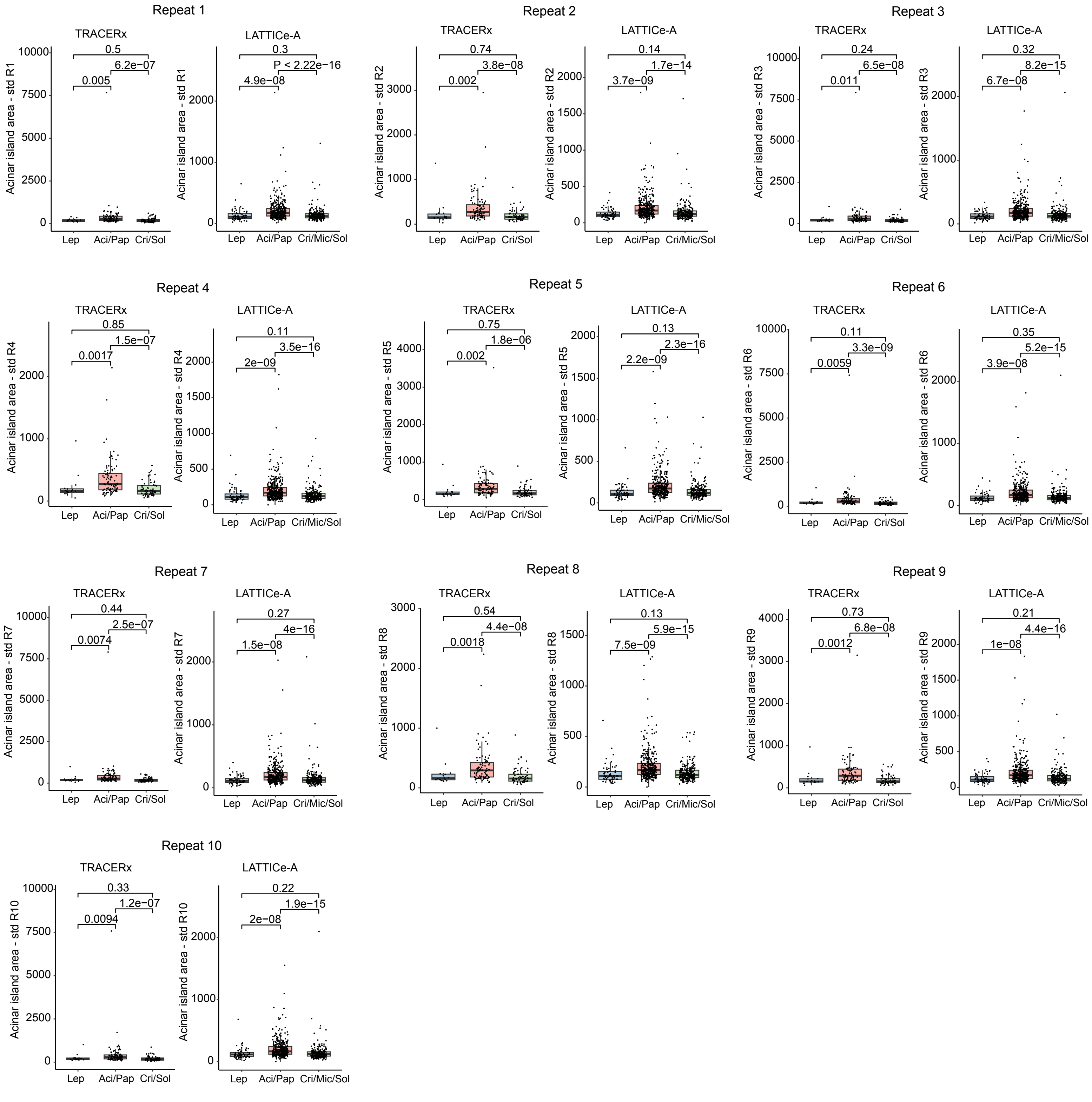

Supplementary Figure 6. Reproducing Extended Data Figure 7c by using 50% of acinar islands per tumor and repeating 10 times. Similar tendency was observed, acinar island areas were less varied in lepidic-predominant (TRACERx 421, P1= 0.005010992, P2=0.002048788, P3=0.010869028, P4=0.001718798, P5=0.002048788, P6=0.005879314, P7= 0.007437248, P8=0.001770160, P9=0.001237530, P100.009355827, n=108; LATTICe-A, P1=4.942281e-08, P2=3.723435e-09, P3=6.715819e-08, P4= 2.031233e-09, P5=2.209578e-09, P6=3.944803e-08, P7=1.524957e-08, P8=7.484980e-09, P9=1.014271e-08, P10=2.040484e-08, n=420) and high-grade-predominant (TRACERx 421, P1=6.229814e-07, P2=3.782820e-08, P3=6.499673e-08, P4=1.481847e-07, P5=1.808808e-06, P6=3.336929e-09, P7=2.536108e-07, P8=4.356487e-08, P9= 6.763151e-08, P10=1.150880e-07, n=157; LATTICe-A, P1<2.22e-16, P2=1.675107e-14, P3=8.158602e-15, P4=3.507372e-16, P5=2.325343e-16, P6=5.159631e-15, P7=3.960935e-16, P8=5.912792e-15, P9=4.400443e-16, P10=1.887863e-15, n=593) tumors than acinar- and papillary-predominant tumors. P value was calculated using a two-sided Wilcoxon rank-sum test and not adjusted for the multiple comparisons. The median value is indicated by a thick horizontal line; the first and third quartiles are represented by box edges; whiskers indicate 1.5 times interquartile range.

Reproduce Extended Data Figure 7d

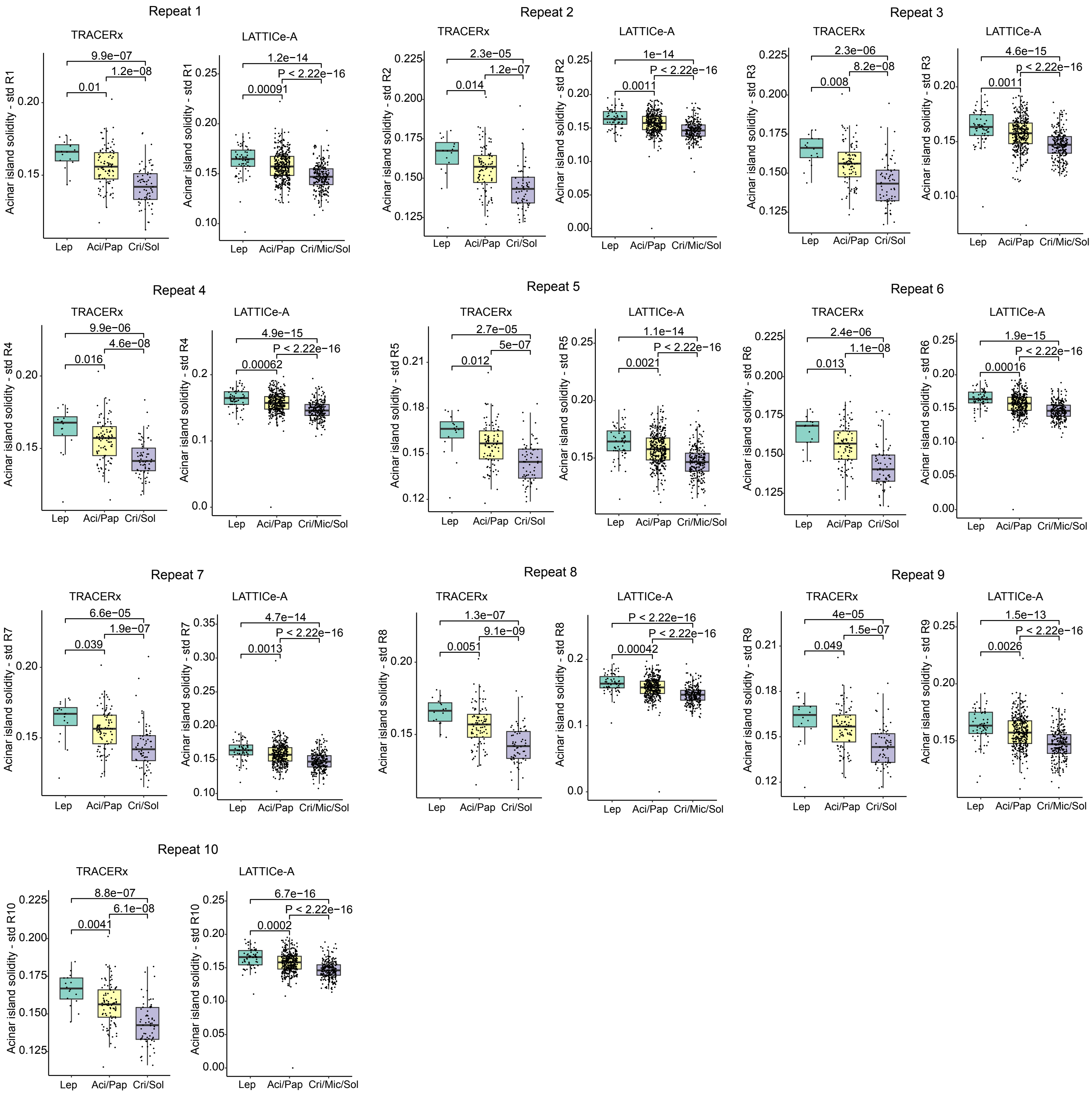

Supplementary Figure 7. Reproducing Extended Data Figure 7d by using 50% of acinar islands per tumor and repeating 10 times. Similar tendency was observed, acinar island shapes were less varied in high-grade-predominant tumors than lepidic predominant tumors (TRACERx 421, P1=9.922079e-07, P2=2.348454e-05, P3=2.277422e-06, P4=9.930203e-06, P5=2.749195e-05, P6=2.414214e-06, P7=6.561608e-05, P8=1.343802e-07, P9=3.951981e-05, P10=8.792449e-07, n=81; LATTICe-A, P1=1.248395e-14, P2=1.037369e-14, P3=4.600002e-15, P4=4.919001e-15, P5=1.079409e-14, P6=1.860518e-15, P7=4.671585e-14, P8<2.22e-16, P9=1.478174e-13, P10=6.654206e-16, n=295). P value was calculated using a two-sided Wilcoxon rank-sum test and not adjusted for the multiple comparisons. The median value is indicated by a thick horizontal line; the first and third quartiles are represented by box edges; whiskers indicate 1.5 times interquartile range.
