# Supplementary material for: The artificial intelligence-based model ANORAK improves histopathological grading of lung adenocarcinoma
Source: Nat Cancer. 2024 Jan 10;5(2):347–63. doi: 10.1038/s43018-023-00694-w (PMC10899116; doi:10.1038/s43018-023-00694-w)
Supplement: Supplementary file 2 — Reporting Summary [file 43018_2023_694_MOESM2_ESM.pdf]

nature portfolio

Corresponding author(s): Yinyin Yuan, John Le Quesne, David A. Moore

Last updated by author(s): Nov 21, 2023

Reporting Summary

Nature Portfolio wishes to improve the reproducibility of the work that we publish. This form provides structure for consistency and transparency in reporting. For further information on Nature Portfolio policies, see our Editorial Policies and the Editorial Policy Checklist.

Statistics

For all statistical analyses, confirm that the following items are present in the figure legend, table legend, main text, or Methods section.

n/a	Confirmed	
☐	☒	The exact sample size (*n*) for each experimental group/condition, given as a discrete number and unit of measurement
☐	☒	A statement on whether measurements were taken from distinct samples or whether the same sample was measured repeatedly
☐	☒	The statistical test(s) used AND whether they are one- or two-sided<br>*Only common tests should be described solely by name; describe more complex techniques in the Methods section.*
☐	☒	A description of all covariates tested
☐	☒	A description of any assumptions or corrections, such as tests of normality and adjustment for multiple comparisons
☐	☒	A full description of the statistical parameters including central tendency (e.g. means) or other basic estimates (e.g. regression coefficient) AND variation (e.g. standard deviation) or associated estimates of uncertainty (e.g. confidence intervals)
☐	☒	For null hypothesis testing, the test statistic (e.g. *F*, *t*, *r*) with confidence intervals, effect sizes, degrees of freedom and *P* value noted<br>*Give P values as exact values whenever suitable.*
☒	☐	For Bayesian analysis, information on the choice of priors and Markov chain Monte Carlo settings
☒	☐	For hierarchical and complex designs, identification of the appropriate level for tests and full reporting of outcomes
☐	☒	Estimates of effect sizes (e.g. Cohen's *d*, Pearson's *r*), indicating how they were calculated

Our web collection on statistics for biologists contains articles on many of the points above.

Software and code

Policy information about availability of computer code

Data collection	Main source of data collection is microscopic H&E scanning. We used the NanoZoomer Digital Pathology System version 3.1.7 (Hamamatsu, Japan) for digitalizing H&E slides.
Data analysis	AI pipeline generating growth pattern mask is available at https://github.com/xi11/AIgrading<br>Python v3.8<br>h5py v2.10.0<br>keras v2.4.3<br>numpy v1.20.3<br>opencv-python v4.5.3.56<br>pandas v1.3.2<br>pillow v8.3.1<br>scipy v1.7.1<br>tensorflow-gpu v2.2.0 for training or inference<br>tensorflow v2.2.0 can also be used for inference<br><br>Statistical analysis code is available at https://github.com/xi11/AIgrading<br>R v4.1.2<br>stats v4.1.2<br>tidyverse v2.0.0<br>irr v0.84.1<br>caret v6.0-93

tidyr v1.3.0
survminer v0.4.9
survival v3.2-13
ggplot2 v3.4.1
ggpubr v0.5.0
survC1 v1.0-3
car v3.0-12
RColorBrewer v1.1-3

For manuscripts utilizing custom algorithms or software that are central to the research but not yet described in published literature, software must be made available to editors and reviewers. We strongly encourage code deposition in a community repository (e.g. GitHub). See the Nature Portfolio guidelines for submitting code & software for further information.

Data

Policy information about availability of data

All manuscripts must include a data availability statement. This statement should provide the following information, where applicable:

- Accession codes, unique identifiers, or web links for publicly available datasets
- A description of any restrictions on data availability
- For clinical datasets or third party data, please ensure that the statement adheres to our policy

The training dataset consisting of annotations on small image tiles are deposited in Zenodo (doi.org/10.5281/zenodo.10016027). Previously published image data that were re-analysed in this study can be requested from https://bmirds.github.io/LungCancer/. The human lung adenocarcinoma diagnostic slide images were derived from the TCGA Research Network: https://portal.gdc.cancer.gov/. Source data for Figures 2, 3, 5b, 5c, 5d, 5e, 5g, 5h, 5i and Extended Data Figures 2, 3a, 5a, 7b, 7c, 7d, 7f, except for clinical data of LATTICe-A cohort, have been provided as Source Data files. Images generated by the AI model in Figure 2a, Extended Data Figures 2, 3a and 7f are deposited in 10.6084/m9.figshare.24599796. For the TRACERx study, all of the scanned diagnostic histological images have a study number label embedded in the file which prevents complete anonymisation. These images cannot therefore be shared, in line with the ethical approval of the study. Request for access to the TRACERx dataset for academic non commercial research purposes can be submitted through the Cancer Research UK and UCL Cancer Trials Centre (ctc.tracerx@ucl.ac.uk), and subject to review of a project proposal that will be evaluated by a TRACERx data access committee, entering into an appropriate data access agreement and any applicable ethical approvals. The timeframe of response to requests is about 6 months. LATTICe-A study data and materials are currently subject to a material and data transfer agreement between University of Leicester, University of Cambridge and NHS Greater Glasgow and Clyde which includes a restricted access period of 5 years precluding any access by other third parties during this time. After the 5 year restricted access data can be accessed by application to NHS Greater Glasgow and Clyde Biorepository (clare.orange@ggc.scot.nhs.uk; john.lequesne@glasgow.ac.uk) as custodians and the data access request will be reviewed and released under their REC approved tissue bank protocols. Requests are reviewed and approved within 6-8 weeks and will be accompanied by a data sharing agreement detailing conditions and restrictions of use and publication.

Human research participants

Policy information about studies involving human research participants and Sex and Gender in Research.

Reporting on sex and gender

Sex and gender were not considered in the study design. For TRACERx 421, self-reported sex was collected and used in the analyses, denoted as 'Sex'. For LATTICe-A, sex assigned at birth was collected and used in the analyses, denoted as 'Sex'.

Population characteristics

Please note that the study started recruiting patients in 2016, when TNM version 7 was standard of care. The up-to-date inclusion/exclusion criteria now utilizes TNM version 8.

TRACERx inclusion and exclusion criteria

Inclusion Criteria:
_Written Informed consent
_Patients ≥18 years of age, with early stage I-IIIB disease (according to TNM 8th edition) who are eligible for primary surgery.
_Histopathologically confirmed NSCLC, or a strong suspicion of cancer on lung imaging necessitating surgery (e.g. diagnosis determined from frozen section in theatre)
_Primary surgery in keeping with NICE guidelines planned
_Agreement to be followed up at a TRACERx site
_Performance status 0 or 1
_Minimum tumor diameter at least 15mm to allow for sampling of at least two tumour regions (if 15mm, a high likelihood of nodal involvement on pre-operative imaging required to meet eligibility according to stage, i.e. T1N1-3)

Exclusion Criteria:
_Any other* malignancy diagnosed or relapsed at any time, which is currently being treated (including by hormonal therapy).
_Any other* current malignancy or malignancy diagnosed or relapsed within the past 3 years**.
*Exceptions are: non-melanomatous skin cancer, stage 0 melanoma in situ, and in situ cervical cancer
**An exception will be made for malignancies diagnosed or relapsed more than 2, but less than 3, years ago only if a pre-operative biopsy of the lung lesion has confirmed a diagnosis of NSCLC.
_Psychological condition that would preclude informed consent
_Treatment with neo-adjuvant therapy for current lung malignancy deemed necessary
_Post-surgery stage IV
_Known Human Immunodeficiency Virus (HIV), Hepatitis B Virus (HBV), Hepatitis C Virus (HCV) or syphilis infection.
_Sufficient tissue, i.e. a minimum of two tumor regions, is unlikely to be obtained for the study based on pre-operative imaging

Patient ineligibility following registration
_There is insufficient tissue
_The patient is unable to comply with protocol requirements
_There is a change in histology from NSCLC following surgery, or NSCLC is not confirmed during or after surgery.
_Change in staging to IIIC or IV following surgery
_The operative criteria are not met (e.g. incomplete resection with macroscopic residual tumors (R2)). Patients with microscopic residual tumors (R1) are eligible and should remain in the study
_Adjuvant therapy other than platinum-based chemotherapy and/or radiotherapy is administered.

The external validation cohort was obtained from the Leicester Archival Thoracic Tumor Investigatory Cohort-Adenocarcinoma (LATTICe-A) study which consists of 845 University Hospitals of Leicester (UHL) Trust patients who underwent surgical treatment with curative intent for primary invasive non-mucinous lung adenocarcinoma. 401 were men and 444 were women with a mean age of 67.66. Most clinical data (age, sex, adjuvant therapy status and time to recurrence or death) were available for all patients, with complete pathological stage for 729 and smoking history for 742. LATTICe-A patients were initially identified using diagnostic histopathology reports from surgical specimens within UHL's local histopathology database.
Inclusion criteria:
• Surgical resection procedures included are wedge resection; lobectomy; bilobectomy; segmentectomy; and pneumonectomy.
• Patients diagnosed with synchronous primary lung tumours, including non-adenocarcinoma tumours were considered eligible for inclusion
• Patients >= 18 years of age.
Exclusion criteria:
• Patients must not have been diagnosed with any other lung tumour in the previous five years before surgical treatment for the primary lung adenocarcinoma.
• Patients who have been diagnosed with metastatic lung adenocarcinoma.
• Lung adenocarcinoma tumours diagnosed as a recurrence of a previous primary lung adenocarcinoma.
• Patients who have only had biopsy surgery performed i.e. core biopsies/EBUS.
• Patients who have been diagnosed with combined tumour types e.g. adenosquamous carcinoma or combined adenocarcinoma/high grade neuroendocrine carcinoma.
• Patients who have been diagnosed with undifferentiated non-small cell lung tumours.

Recruitment

Patients seen with a new diagnosis of lung cancer in lung cancer units across the United Kingdom, according to the eligibility criteria above, were recruited. No selection bias has been identified to date.
For TRACERx, clinical and pathological data is collected from patients during study follow up - this period is a minimum of five years. Data collection is overseen by the sponsor of the study (Cancer Research UK & UCL Cancer Trials Centre) and takes place in 19 hospitals across the United Kingdom. A centralised database called MACRO is used for this purpose. Recruitment started in April 2014 and is still ongoing in London and Manchester. Survival data last updated in 15 June 2021. LATTICe-A, the Leicester Archival Thoracic Tumor Investigatory Cohort – Adenocarcinoma, is a continuous retrospective series of resected primary LUAD tumors from a single surgical center, University Hospitals of Leicester (UHL) Trust, between 1998 and 2014.
In TRACERx, disease-free survival (DFS) was measured from the time of study registration to date of first lung recurrence or death from any cause. Patients who didn't have these events were censored at the date last known to be alive (including patients who developed a new primary tumor that has been shown biologically not to be linked to the initial primary lung tumor). Overall survival was measured from the time of study registration to date of death from any cause.
In the LATTICE-A cohort, recurrence data were obtained from examination of patient records, notably paper notes and radiological databases, to identify the date of radiologically or biopsy-confirmed recurrence. Cancer-specific death is determined by the presence of lung cancer in the cause of death in the death certificate. Overall survival refers to the date of death.

Ethics oversight

The study was approved by the NRES Committee London with the following details:
Study title: TRAcking non small cell lung Cancer Evolution through therapy (Rx)
REC reference: 13/LO/1546
Protocol number: UCL/12/0279
IRAS project ID: 138871
Study protocol: https://clinicaltrials.gov/ct2/show/NCT01888601

The LATTICe-A cohort was ethically approved by an NHS research ethics committee (ref. 14/EM/1159), more information can be found at: https://www.hra.nhs.uk/planning-and-improving-research/application-summaries/researchsummaries/characterisation-of-thoracic-malignancies-using-archival-human-tissue/

Note that full information on the approval of the study protocol must also be provided in the manuscript.

Field-specific reporting

Please select the one below that is the best fit for your research. If you are not sure, read the appropriate sections before making your selection.

☒ Life sciences ☐ Behavioural & social sciences ☐ Ecological, evolutionary & environmental sciences

For a reference copy of the document with all sections, see nature.com/documents/nr-reporting-summary-flat.pdf

Life sciences study design

All studies must disclose on these points even when the disclosure is negative.

Sample size	No statistical methods were used to predetermine sample size. Primary tumours diagnosed as invasive non-mucinous lung adenocarcinoma from 206 patients were analysed for TRACERx 421. The sample size is similar to the one reported in a previous publication (PMID: 37045996, 37046096) and subject to available diagnostic slides. Primary tumours diagnosed as invasive non-mucinous lung adenocarcinoma from 845 patients were analysed for LATTICe-A cohort. The sample size is similar to the one reported in a previous publication (PMID: 32461698). Primary tumours diagnosed as invasive non-mucinous lung adenocarcinoma from 178 patients were analysed for TCGA, with a similar samples size reported in a previous publication (PMID: 25079552) and subject to available diagnostic slides. The sample size, 143, in DHMC is as same as the one reported in a previous publication (PMID: 30833650). The objective of this study is to validate the performance of AI model against manual scorings, compared with similar studies (PMID: 30833650, 30728398), 1372 cases from four independent cohorts are sufficient.
Data exclusions	Please see study inclusion/exclusion criteria above. Additionally, tumours diagnosed as mucinous lung adenocarcinoma, adenocarcinoma in situ, minimally invasive adenocarcinomas and other variants were excluded.
Replication	The AI model developed for TRACERx 100 was applied directly to TRACERx 421, LATTICe-A, TCGA and DHMC cohorts, achieving a degree of agreement with human pathologists equivalent to inter-pathologist agreement and allowing the replication of the prognostic value of AI grading in TRACERx 421 and LATTICe-A cohorts.
Randomization	Randomization is not relevant as this is an observational study.
Blinding	Blinding is not relevant as this is an observational study. Patients were not allocated to any interventions and they were followed up and assessed as per routine practice. No results from this study are reported back to patients, so there is no likelihood of people changing their behaviors based on these findings. The deep learning model was training without knowing the outcome of patients, which represents a form of blinding.

Reporting for specific materials, systems and methods

We require information from authors about some types of materials, experimental systems and methods used in many studies. Here, indicate whether each material, system or method listed is relevant to your study. If you are not sure if a list item applies to your research, read the appropriate section before selecting a response.

Materials & experimental systems

n/a	Involved in the study	
☒	☐	Antibodies
☒	☐	Eukaryotic cell lines
☒	☐	Palaeontology and archaeology
☒	☐	Animals and other organisms
☒	☐	Clinical data
☒	☐	Dual use research of concern

Methods

n/a	Involved in the study	
☒	☐	ChIP-seq
☒	☐	Flow cytometry
☒	☐	MRI-based neuroimaging
